# Artemyrianosins A–J, cytotoxic germacrane-type sesquiterpene lactones from *Artemisia myriantha*

**DOI:** 10.1007/s13659-022-00340-5

**Published:** 2022-05-02

**Authors:** Xin Zhang, Yun-Bao Ma, Xiao-Feng He, Tian-Ze Li, Chang-An Geng, Li-Hua Su, Shuang Tang, Zhen Gao, Ji-Jun Chen

**Affiliations:** 1 State Key Laboratory of Phytochemistry and Plant Resources in West China, Yunnan Key Laboratory of Natural Medicinal Chemistry, Kunming Institute of Botany, Chinese Academy of Sciences, 132# Lanhei Road, Kunming, 650201 Yunnan People’s Republic of China; 2 University of Chinese Academy of Sciences, Beijing, 100049 People’s Republic of China

**Keywords:** *Artemisia myriantha*, Artemyrianosins A–J, Germacrane-type sesquiterpenoids, Cytotoxicity

## Abstract

Ten new germacrane-type sesquiterpenoids, artemyrianosins A–J (**1**–**10**), were isolated from the aerial parts of *Artemisia myriantha*. Their structures were elucidated by spectral analyses including UV, IR, HRESIMS, 1D and 2D NMR, ECD and the absolute configurations of compounds **1** and **7**–**9** were characterized using X-ray crystallography. All isolates were tested their cytotoxicity against three human hepatoma cell lines (HepG2, Huh7, and SK-Hep-1), and compounds **1**–**3**, **7**, and **10** showed cytotoxicity with IC_50_ values ranging from 43.7 to 89.3 μM. Among them, the most active compound **3** exhibited activity against three human hepatoma cell lines with IC_50_ values of 43.7 μM (HepG2), 47.9 μM (Huh7), and 44.9 μM (SK-Hep-1).

**Graphical Abstract:**

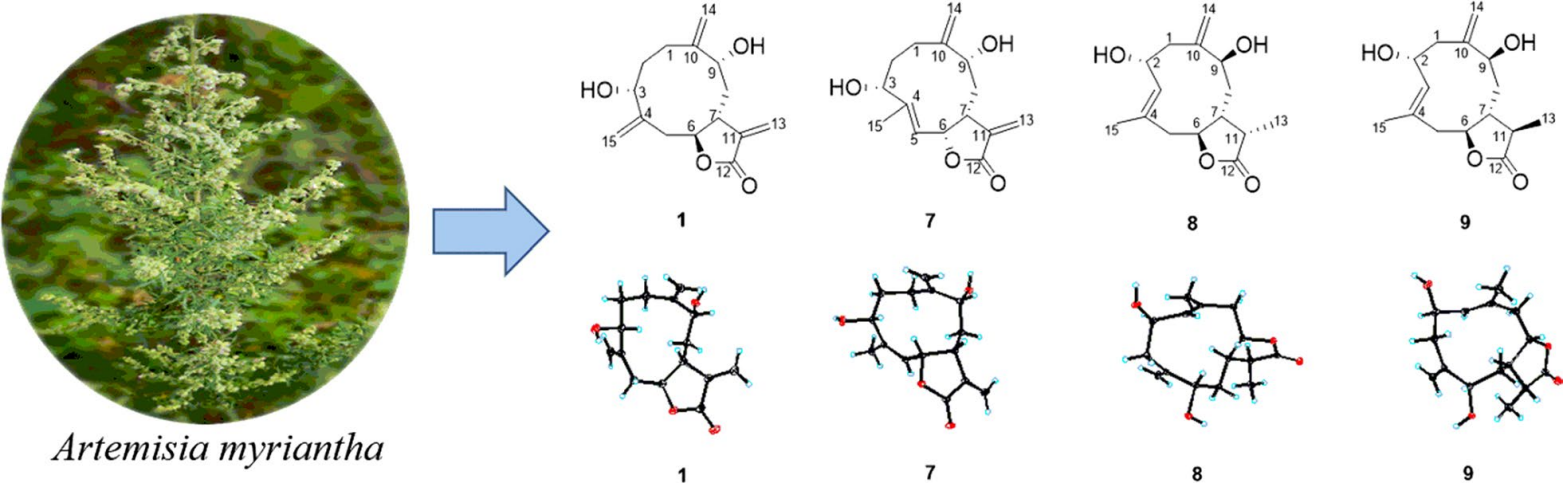

**Supplementary Information:**

The online version contains supplementary material available at 10.1007/s13659-022-00340-5.

## Introduction

**Fig. 1:**
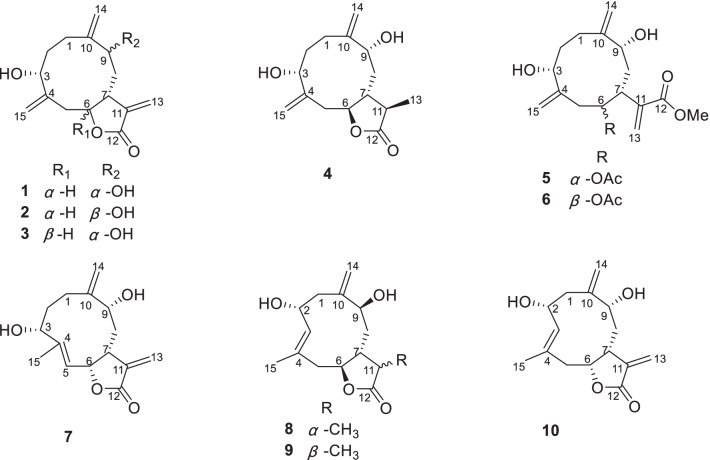
Chemical structures of compounds **1**–**10**

Hepatocellular carcinoma (HCC) as one of the most serious and common type of liver cancer is mainly caused by HBV or HCV infection, and heavy alcohol intake [1, 2]. HCC has resulted in nearly 0.83 million deaths worldwide in the year 2020 [3, 4], and suffered more than 1 million people will be affected by 2025 [5]. Clinically, four synthetic ones (sorafenib, regorafenib, lenvatinib and cabozantinib) and three monoclonal antibody ones (nivolumab, pembrolizumab and ramucirumab) are used to treat HCC [6, 7]. Although these drugs have obtained significant achievements, there are still some inevitable drawbacks, such as the low objective response rate, high incidence of adverse reactions, and drug resistance [8]. Therefore, new drugs with different targets for treating HCC are urgently needed (Fig. 1).

*Artemisia*, one of the dominant genus within Asteraceae family, contains nearly 380 species globally with 186 species being dispersed in China [9]. Many *Artemisia* plants, such as *A. annua*, *A. argyi*, *A*. *capillaris*, and *A. scoparia*, have been recorded for the treatment of malaria, inflammation, hepatitis, cancer in the traditional Chinese medicine system [9–12]. Phytochemical investigation revealed that *Artemisia* genus are rich in sesquiterpenoids with antimalarial, antiinflammatory, antitumor, cytotoxic, antibacterial, and antihelminthic activities [13]. For example, artemisinin, a sesquiterpenoid lactone with an unusual peroxide bridge, which was obtained from *A. annua* by the Chinese scientist You-You Tu in 1972, showed antimalarial, anticancer and antiinflammatory activities [14]. Dihydroartemisinin, artemether, and artesunate which were chemically modified from artemisinin also exhibited antimalarial, antiviral, antifungal, anticancer, and antiinflammatory properties [15]. Arglabin, a guaiane-type sesquiterpenolide from *A. glabella*, inhibited of farnesyltransferase and its dimethylamino hydrochloride has been successfully developed into an anticancer drug in Kazakhstan for the treatment of colon, breast, ovarian, lung and liver cancers [16]. Arteminolides A–D from *A. argyi* were potential inhibitors of farnesyl protein transferase (FPTase) with IC_50_ values less than 1.0 μM in vitro, of which arteminolide C could prevent the development of lung tumor and human colon xenograft without causing weight loss in nude mice [17].

Our ongoing efforts to investigate bioactive sesquiterpenoids from the *Artemisia* plants, bioassay-guided isolation of *A. atrovirens* let to 26 guaiane dimers ([4 + 2] Diels–Alder cyclization) [18, 19], six rotundane-guaiane dimers ([4 + 2] Diels–Alder cyclization or containing a methylene-bridge) [19, 20], two guaiane-rotundane-guaiane trimers (containing a methylene-bridge) [20], two novel cagelike sesquiterpenoids (formed by intramolecular Diels–Alder cycloaddition) [21], and 16 undescribed guaiane sesquiterpenoids [9]. Among them, four guaiane-guaiane dimers (lavandiolide H and artematrolides A, J, and K) possessed significant cytotoxicity against HepG2, SMMC-7721, and Huh7 cell lines with IC_50_ values ranging from 3.8 to 9.6 μM, and lavandiolide H could induce G2/M cell cycle arrest and apoptosis in HepG2 cells via upregulating cleaved-PARP-1 and downregulating BCL-2 and PARP-1 [18]. Furthermore, artematrolide A was shown to activate the ROS/ERK/mTOR signaling pathway and promote metabolic shift in cervical cancer cells [22]. And the biomimetic synthesis via Diels–Alder reaction of the guaianolide dimers (artematrolide F and lavandiolides H, I, and K) and a battery of analogues were also achieved [23].

*A. myriantha* Wall. ex Bess. is commonly used for treating menorrhagia and inflammatory diseases in traditional Chinese medicine [24]. Phytochemical investigation on this species revealed the presence of sesquiterpenoids, flavonoids, and essential oils [25]. Among them, some sesquiterpenoids showed cytotoxicity against human colon cancer HCT-8, human gastric cancer BGC-823, and human liver cancer Bel-7402 cells [26, 27]. Our previous investigation reported 23 undescribed sesquiterpenolides with cytotoxicity against HepG2, SMMC-7721, and Huh7 from *A. myriantha*, classifying as germacranolide, guaianolide, and eudesmanolide, and revealed that artemyrianolide H displayed promising cytotoxicity against HepG2, SMMC-7721, and Huh7 with IC_50_ values of 4.9, 3.1, and 4.3 μM, respectively [25]. During our continuous search for antihepatic sesquiterpenoids from *A. myriantha*, 10 undescribed germacranolides (**1**‒**10**) were discovered (Fig. 1). Hence their isolation, structural identification, and cytotoxicity were discussed in this study.

## Results and discussion

**Fig. 2:**
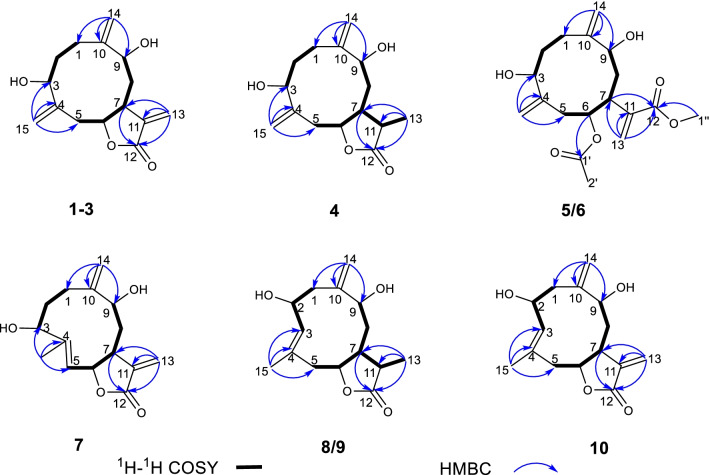
Key ^1^H-^1^H COSY and HMBC correlations of compounds **1**–**10**

**Fig. 3:**
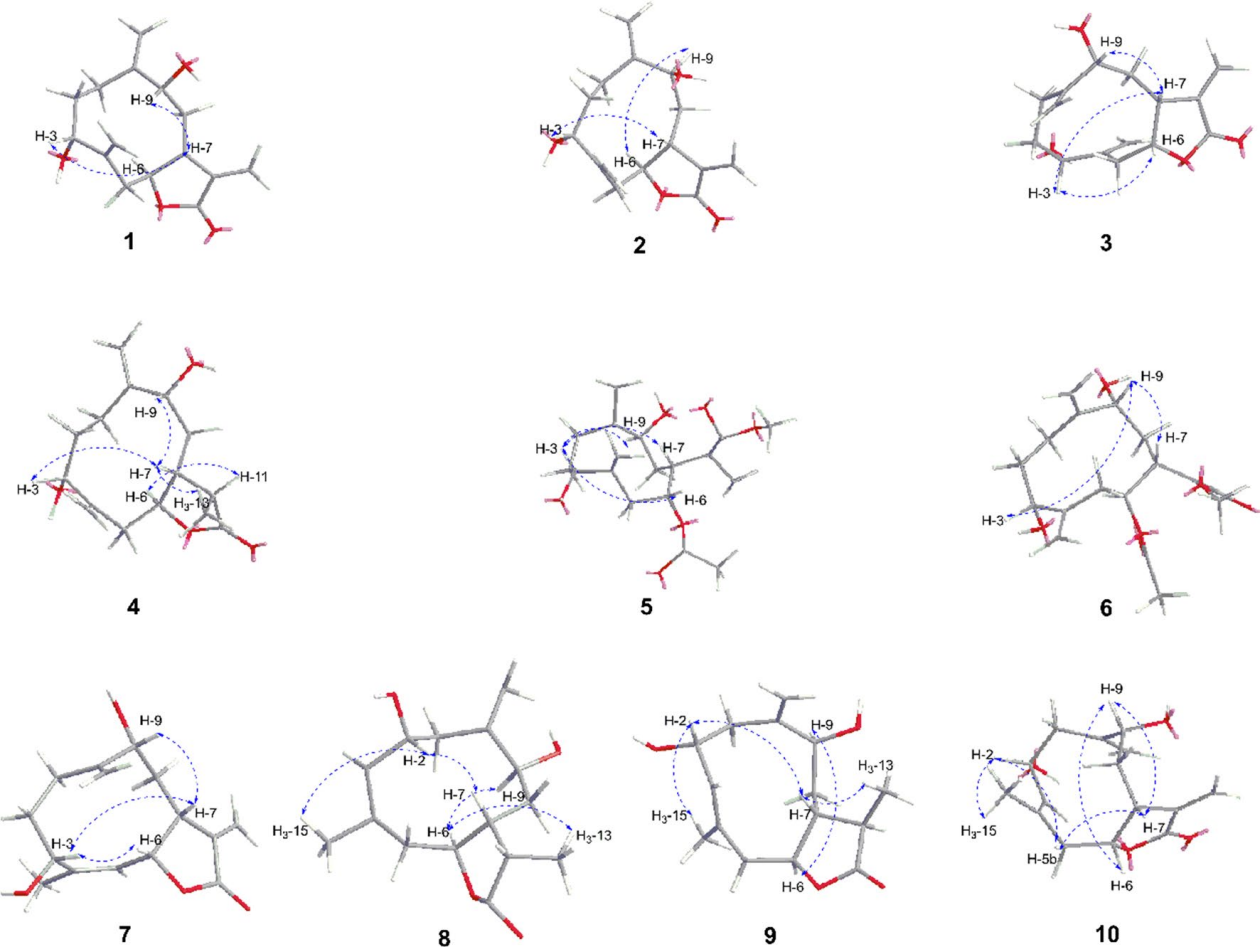
Key ROESY correlations of compounds **1**–**10**

**Fig. 4:**
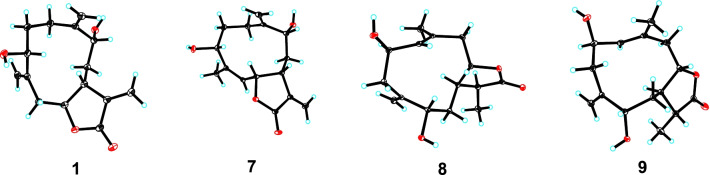
The X-ray ORTEP drawings of compounds **1** and **7**–**9**

Artemyrianosin A (**1**) showed a molecular formula of C_15_H_20_O_4_ based on the analysis of the (+)-HRESIMS ion at *m/z* 287.1254 [M + Na]^+^ (calcd for C_15_H_20_O_4_Na, 287.1254) with six degrees of unsaturation. Its IR spectrum exhibited the presence of hydroxy (3414 cm^‒1^), carbonyl (1757 cm^‒1^), and double-bond (1643 and 1570 cm^‒1^) groups. The ^1^H and ^13^C NMR data (Tables 1 and 2) resembled those of artemyrianolide M [25], except for the only difference being that a ketone group at C-3 in artemyrianolide M was replaced by one oxygenated methine [*δ*_H_ 4.11 (1H, dd, *J* = 12.7, 5.1 Hz, H-3), *δ*_C_ 73.3 (C-3)]. This deduction was confirmed by the HMBC correlations from H-3 to C-15 (*δ*_C_ 117.2) and C-4 (*δ*_C_ 144.2) as well as the correlations of H-1/H_2_-2/H-3 in the ^1^H-^1^H COSY spectrum (Fig. 2). To determine its relative configuration, a ROESY experiment was carried out. The cross-peaks of H-3/H-7 and H-7/H-9 in the ROESY spectrum (Fig. 3) indicated that these protons were cofacial and *β*-oriented. However, the correlations of H-6/H-9 or H-6/H-3 were not observed in the ROESY spectrum, suggesting that H-6 was *α*-oriented. The absolute configuration of **1** was unambiguously verified to be (3*R*,6*S*,7*R*,9*R*) by Cu K*α* X-ray crystallographic analysis (Fig. 4). Therefore, the structure of compound **1** was defined as (3*R*,6*S*,7*R*,9*R*)-3,9-dihydroxygermacra-4(15),10 (14),11(13)-trien-12,6-olide.

**Table 1:** ^1^H NMR data for compounds **1**–**5** (600 MHz, CD_3_OD, *δ* in ppm, *J* in Hz)

No	**1**	**2**	**3**	**4**	**5**
1a	2.13, m	2.24, m	2.27, m	2.30, m	2.31, m
1b		1.97, m	2.06, m		
2a	2.10, m	2.14, m	2.16, m	2.18, m	2.04, m
2b		1.95, m	1.92, m	1.90, m	1.91, m
3	4.11, dd (10.9, 3.1)	4.18, dd (10.6, 4.4)	4.35, dd (9.2, 5.3)	4.04, dd (8.8, 4.1)	4.23, dd (10.2, 4.0)
5a	3.05, dd (12.7, 5.1)	3.02, dd (13.0, 4.7)	2.58, m	2.37, m	2.37, m
5b	2.07, m	2.06, m	2.45, dd (15.5, 10.7)		
6	4.30, m	4.37, ddd (11.0, 6.2, 4.8)	4.93, ddd (10.6, 7.5, 3.2)	4.90, m	5.35, td (8.0, 2.4)
7	2.58, m	2.93, m	3.51, m	2.38, m	3.67, m
8a	1.94, m	2.03, m	2.38, m	2.07, m	2.27, m
8b	1.83, m	1.93, m	2.00, m	1.79, m	1.91, m
9	4.28, m	4.23, m	4.17, d (3.6)	4.16, dd (7.8, 6.1)	4.36, dd (6.3, 2.6)
11				2.36, m	
13a	6.15, d (3.4)	6.20, dd (3.0, 0.6)	6.19, d (3.1)	1.19, d (6.4)	6.30, s
13b	5.75, d (3.4)	6.11, dd (3.0, 0.6)	5.69, d (3.1)		5.73, s
14a	5.26, s	5.34, d (1.4)	5.44, s	5.12, s	5.13, s
14b	5.20, s	5.16, s	5.08, s	4.93, s	5.04, s
15a	5.37, s	5.33, s	5.20, s	5.37, s	5.22, s
15b	5.33, s	5.23, s	5.15, s	5.18, s	5.19, s
2′					1.98, s
OMe					3.75, s

**Table 2:** ^13^C NMR data for compounds **1**–**10** (150 MHz, CD_3_OD, *δ* in ppm)

No	**1**	**2**	**3**	**4**	**5**	**6**	**7**	**8**	**9**	**10**
1	23.4, CH_2_	28.0, CH_2_	29.9, CH_2_	26.6, CH_2_	31.6, CH_2_	24.3, CH_2_	23.7, CH_2_	43.5, CH_2_	43.0, CH_2_	43.1, CH_2_
2	32.5, CH_2_	32.6, CH_2_	31.6, CH_2_	34.1, CH_2_	31.6, CH_2_	34.4, CH_2_	28.6, CH_2_	70.8, CH	70.4, CH	70.4, CH
3	73.3, CH	74.4, CH	70.4, CH	75.7, CH	77.2, CH	76.4, CH	66.8, CH	132.5, CH	132.6, CH	132.0, CH
4	144.2, C	145.3, C	147.8, C	151.6, C	146.8, C	147.6, C	143.8, C	133.5, C	132.9, C	133.0, C
5	41.3, CH_2_	40.8, CH_2_	37.0, CH_2_	34.1, CH_2_	33.0, CH_2_	35.7, CH_2_	123.8, C	41.1, CH_2_	43.0, CH_2_	42.8, CH_2_
6	85.2, CH	85.0, CH	81.8, CH	83.9, CH	74.4, CH	74.7, CH	78.5, CH	82.8, CH	83.3, CH	83.4, CH
7	41.0, CH	40.3, CH	39.0, CH	39.9, CH	35.5, CH	37.5, CH	42.5, CH	38.0, CH	43.7, CH	39.9, CH
8	40.5, CH_2_	40.2, CH_2_	32.6, CH_2_	36.3, CH_2_	32.8, CH_2_	31.6, CH_2_	33.5, CH_2_	31.2, CH_2_	38.7, CH_2_	41.2, CH_2_
9	77.6, CH	72.3, CH	72.2, CH	76.6, CH	72.1, CH	77.2, CH	78.8, CH	74.2, CH	73.4, CH	73.3, CH
10	149.9, C	150.7, C	150.3, C	151.5, C	149.1, C	150.8, C	149.6, C	147.0, C	147.8, C	146.8, C
11	143.4, C	142.6, C	141.8, C	46.3, CH	144.3, C	142.2, C	139.8, C	38.6, CH	44.2, CH_2_	141.8, C
12	171.5, C	172.2, C	172.2, C	181.2, C	169.3, C	169.0, C	172.3, C	182.6, C	182.2, C	172.2, C
13	121.4, CH_2_	124.6, CH_2_	121.3, CH_2_	13.5, CH_3_	126.4, CH_2_	126.2, CH_2_	121.6, CH_2_	10.9, CH_3_	16.4, CH_3_	124.7, CH_2_
14	115.9, CH_2_	112.2, CH_2_	111.7, CH_2_	113.4, CH_2_	114.0, CH_2_	116.1, CH_2_	113.4, CH_2_	114.1, CH_2_	113.7, CH_2_	113.6, CH_2_
15	117.2, CH_2_	118.1, CH_2_	116.2, CH_2_	112.4, CH_2_	117.2, CH_2_	115.3, CH_2_	16.7, CH_3_	20.8, CH_3_	21.0, CH_3_	22.1, CH_3_
1′					172.2, C	172.3, C				
2′					21.0, CH_3_	20.9, CH_3_				
1″'					52.4, CH_3_	52.5, CH_3_

**Fig. 5:**
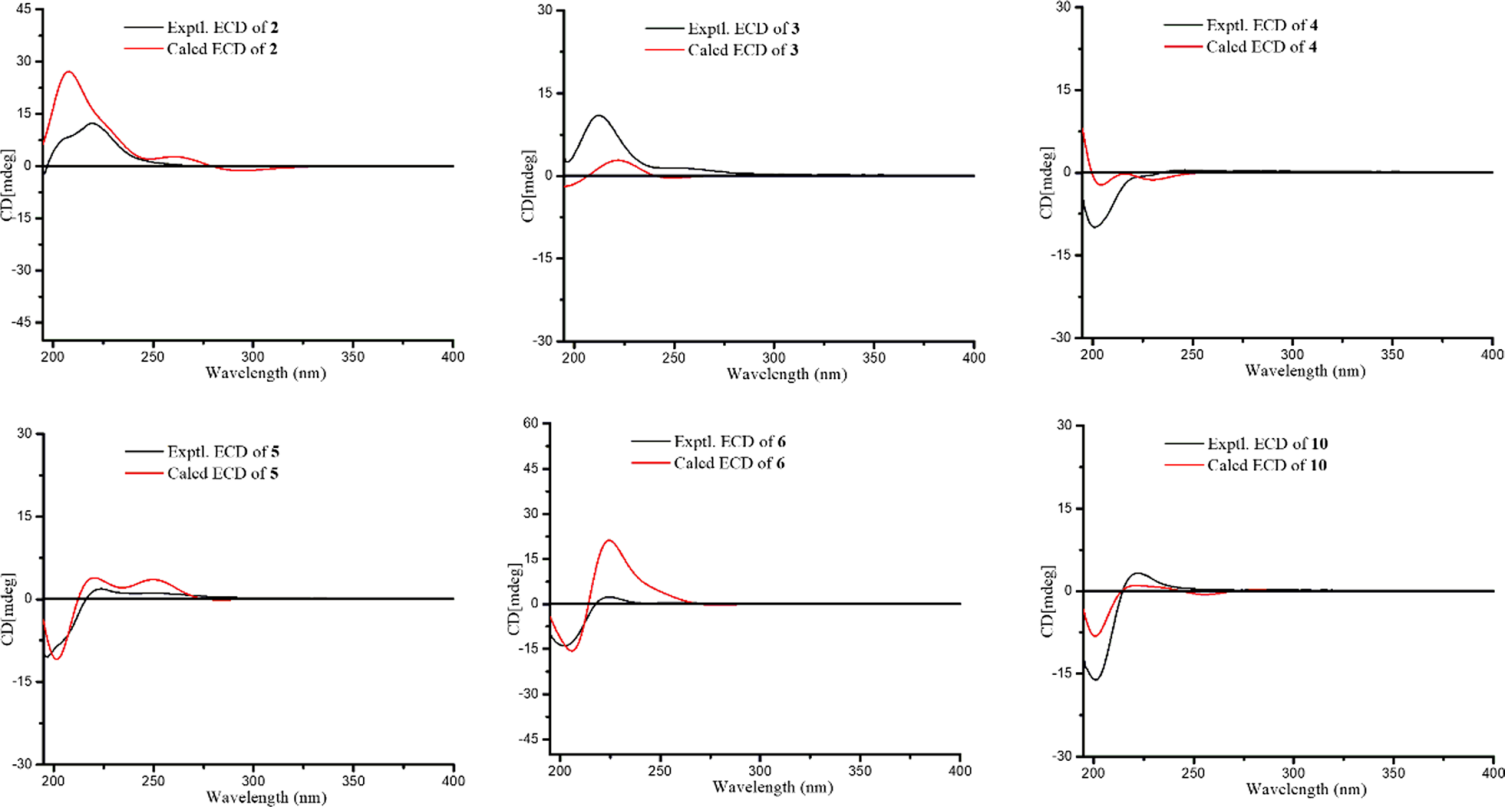
The experimental and calculated ECD spectra of compounds **2–6**, and **10**

Artemyrianosin B (**2**) was deduced with the same molecular formula of C_15_H_20_O_4_ as **1** by the HRESIMS at *m/z* 265.1427 [M + H]^+^ (calcd for C_15_H_21_O_4_, 265.1434). The ^1^H and ^13^C NMR data (Tables 1 and 2) of compound **2** were closely related to those of **1**, and further analyses of 2D NMR spectra implied that they possessed the same planar structure. Their only difference was the chemical shift changes of H-9 (*δ*_H_ 4.23 *vs* 4.28) and C-9 (*δ*_C_ 72.3 *vs* 77.6) and those surrounding the C-9 position (Tables 1 and 2), which might be caused by the different configurations at C-9. In the ROESY spectrum (Fig. 3), the correlations of H-3/H-7 and H-6/H-9 revealed *β*-orientations of H-3 and H-7, whereas *α*-orientations of H-6 and H-9. Its absolute configuration was assigned to be 3*R*,6*S*,7*R*,9*S* by comparison of its experimental ECD spectrum with the calculated one (Fig. 5). Therefore, the structure of compound **2** was established as (3*R*,6*S*,7*R*,9*S*)-3,9-dihydroxygermacra-4 (15), 10 (14), 11 (13)-trien-12,6-olide (Fig. 5).

Artemyrianosin C (**3**) was assigned to have the same molecular formula C_15_H_20_O_4_ with compounds **1** and **2** from the HRESIMS at *m/z* 265.1431 [M + H]^+^ (calcd for C_15_H_21_O_4_, 265.1434). Compound **3** had the identical 2D structure as **1** and **2** based on their 1D and 2D NMR data (Tables 1 and 2, Fig. 2). The ROESY correlations of H-3/H-6, H-3/H-7, and H-7/H-9 revealed that these protons were *β*-oriented. Its absolute configuration was concluded to be 3*R*,6*R*,7*R*,9*R* by comparison of its experimental ECD spectrum with the calculated one (Fig. 5). Thus, the structure of compound **3** was established to be (3*R*,6*R*,7*R*,9*R*)-3,9-dihydroxygermacra-4 (15),10(14),11(13)-trien-12,6-olide.

Artemyrianosin D (**4**) had a molecular formula C_15_H_22_O_4_ according to the HRESIMS data at *m/z* 267.1579 [M + H]^+^ (calcd for C_15_H_23_O_4_, 267.1591), suggesting five degrees of unsaturation. The similarity of ^1^H and ^13^C NMR data (Tables 1 and 2) between compounds **4** and **1** indicated that they were structural analogs, and the main difference was that the exocyclic double bond between C-11 and C-13 in compound **1** was disappeared, and a doublet methyl [*δ*_H_ 1.19 (3H, d, *J* = 6.4 Hz, H-13), *δ*_C_ 13.5 (C-13)] and a methine [*δ*_H_ 2.36 (1H, m, H-11), *δ*_C_ 46.3 (C-11)] signals were appeared in compound **4**. This deduction was confirmed by the ^1^H-^1^H COSY interactions of H-7/H-11/H_3_-13 and the HMBC correlations from H_3_-13 to C-7, C-11, and C-12. Its absolute stereochemistry was defined to be (3*R*,6*S*,7*R*,9*R*,11*R*) by the ROESY correlations of H-3/H-7, H-7/H-9, H-7/H-13, and H-6/H-11 and the similarity between the experimental and calculated ECD spectra. Consequently, the structure of compound **4** was assigned as (3*R*,6*S*,7*R*,9*R*,11*R*)-3,9-dihydroxygermacra-4 (15),10(14)-dien-12,6-olide.

Artemyrianosin E (**5**) was determined to possess a molecular formula of C_18_H_26_O_6_ with six indices of hydrogen deficiency by its HRESIMS ion at *m/z* 339.1792 [M + H]^+^ (calcd for C_18_H_27_O_6_, 339.1802). The IR spectrum of **5** showed the characteristic absorptions for hydroxy (3428 cm^−1^), carbonyl (1718 cm^−1^), and olefinic (1631 cm^−1^) functionalities. The ^1^H NMR spectrum (Table 1) of **5** displayed a methyl at *δ*_H_ 1.98 (3H, s), a methoxy *δ*_H_ 3.75 (3H, s), three oxygenated methine protons at *δ*_H_ 5.35 (1H, td, *J* = 8.0, 2.4 Hz, H-6), 4.36 (1H, dd, *J* = 6.3, 2.6 Hz, H-9), and 4.23 (1H, dd, *J* = 10.2, 4.0 Hz, H-3), and three pairs of olefinic methylene protons [*δ*_H_ 6.30 (1H, s, H-13a), 5.73 (1H, s, H-13b); 5.13 (1H, s, H-14a), 5.04 (1H, s, 14b); 5.22 (1H, s, H-15a), 5.19 (1H, s, 15b)]. The ^13^C NMR spectrum (Table 2) showed the existence of 18 carbons, including two methyl groups, seven methylenes (three terminal double bonds, and four aliphatic methylenes), four methines (three oxygenated and an aliphatic methine), and five quaternary carbons (two ester carbonyl and three olefinic carbons). The above characteristic signals indicated that compound **5** was a germacranolide-type sesquiterpenoid with acetoxy and methoxy groups. The above inference was supported by two proton spin systems of H_2_-1/H_2_-2/H-3 and H_2_-5/H-6/H-7/H_2_-8/H-9 in the ^1^H-^1^H COSY spectrum, as well as the HMBC correlations from H_2_-14 to C-1, C-9 and C-10 and from H_2_-15 to C-3, C-4 and C-5. In addition, the correlation from H-6 to C-1′ in the HMBC spectrum implied that the acetoxy group (*δ*_H_ 1.98; *δ*_C_ 172.2 and 21.0) was linked at C-6; the HMBC correlation of OMe to C-12 suggested that the methoxy group was pointed at C-12. In the ROESY spectrum (Fig. 3), the cross peaks of H-3/H-6, H-3/H-7, and H-7/H-9 determined its relative configuration. The absolute configuration of **5** was defined as (3*R*,6*R*,7*R*,9*R*) by the similarity between the experimental and calculated ECD curves. Therefore, the structure of compound **5** was established as (3*R*,6*R*,7*R*,9*R*)-6-acetoxy-3,9-dihydroxygermacra-4(15),10 (14),11(13)-trien-12-oic acid methyl ester.

Artemyrianosin F (**6**) was assigned to have the same molecular formula C_18_H_26_O_6_ with compound **5** from the HRESIMS data at *m/z* 339.1785 [M + H]^+^ (calcd for C_18_H_27_O_6_, 339.1802). The ^1^H and ^13^C NMR data (Tables 2 and 3) of compound **6** were very similar to those of **5**, indicating that both were architecturally semblable. Further analyses of their 2D NMR spectra indicated that the planar structure of compound **6** was identical with that of compound **5**. In the ROESY spectrum, the correlations of H-3/H-9 and H-7/H-9 indicated the homolateral orientation of H-3, H-7, and H-9. However, no correlation of H-6 with the former three protons was observed, but the correlation of H-6/H_2_-8 was obviously appeared in the ROESY spectrum, suggesting that H-6 was on the opposite side. Hence, the absolute stereochemistry of compound **6** was elucidated and named as (3*R*,6*S*,7*R*,9*R*)-6-acetoxy-3,9-dihydroxygermacra-4(15),10(14),11(13)-trien-12-oic acid methyl ester by comparing the calculated and experimental ECD curves.

**Table 3:** ^1^H NMR data for compounds **6**–**10** (600 MHz, CD_3_OD, *δ* in ppm, *J* in Hz)

No	**6**	**7**	**8**	**9**	**10**
1a	2.34, m	2.12, m	2.73, m	2.70, dd (12.7, 5.9)	2.76, dd (12.5, 6.5)
1b	2.19, m	1.70, m	1.93, m	1.98, m	1.99, m
2a	2.29, m	2.20, m	4.54, td (10.1, 5.9)	4.54, td (10.3, 6.0)	4.60, m
2b	1.89, m	1.72, m			
3	3.99, dd (8.9, 2.9)	4.79, dd (10.7, 5.8)	5.27, d (9.7)	5.34, d (8.1)	5.34, d (9.6)
5a	2.63, m	4.98, d (10.6)	2.77, m	2.89, dd (13.2, 6.3)	2.98, dd (13.6, 6.3)
5b	2.16, m		2.05, dd (13.7, 8.0)	1.94, m	1.71, m
6	5.34, ddd (10.1, 5.5, 1.9)	5.50, dd (10.9, 8.2)	4.44, q (6.8)	4.42, m	4.56, m
7	3.29, m	3.11, m	2.27, m	1.86, m	2.68, m
8a	2.04, m	2.04, m	1.82, m	1.88, m	2.03, m
8b	1.81, m	1.83, m	1.61, m		1.91, m
9	3.88, dd (11.7, 4.2)	4.16, dd (11.1, 4.4)	4.10, dd (8.8, 2.9)	4.09, t (5.0)	4.05, dd (9.6, 3.1)
11			2.85, m	2.40, m	
13a	6.35, s	6.24, d (3.7)	1.18, d (7.6)	1.32, d (7.3)	6.24, d (2.8)
13b	5.84, s	5.67, d (3.7)			5.98, d (2.8)
14a	5.01, s	5.33, d (2.7)	5.16, s	5.22, s	5.27, s
14b	4.84, s	5.07, d (2.7)	5.12, s	5.09, s	5.10, s
15a	5.43, s	1.75, d (1.3)	1.74, s	1.78, s	1.73, s
15b	5.22, s				
2′	1.97, s				
OMe	3.76, s				

Artemyrianosin G (**7**) was determined to have a molecular formula of C_15_H_20_O_4_ based on the HRESIMS data at *m/z* 265.1423 [M + H]^+^ (calcd for C_15_H_21_O_4_, 265.1434). The ^1^H and ^13^C NMR data (Tables 2 and 3) of compound **7** resembled to those of **1**, the main difference was that the exocyclic double bond between C-4 and C-15 in **1** was absent and replaced by a trisubstituent double bond [*δ*_H_ 4.98 (1H, d, *J* = 10.6 Hz, H-5); *δ*_C_ 143.8 (C-4) and 123.8 (C-5)] and a singlet methyl [*δ*_H_ 1.75 (3H, d, *J* = 1.3 Hz, H-15); *δ*_C_ 16.7 (C-15)] in **7**. This deduction was supported by the proton spin systems of H-5/H-6/H-7/H_2_-8/H-9 in the ^1^H-^1^H COSY spectrum and the HMBC correlations from H_3_-15 to C-3/C-4/C-5. Its relative configuration was proposed by the ROESY correlations of H-3/H-6 and H-7/H-9. In addition, the ROESY correlation of H-5 with H_3_-15 manifested that *Δ*^4,5^-double bond was *Z*-configuration. The absolute configuration of **7** was defined as 3*R*,6*R*,7*R*,9*R* by a single crystal X-ray crystallographic diffraction experiment with Cu K*α* radiation (Fig. 4). Therefore, the structure of **7** was identified as (3*R*,6*R*,7*R*,9*R*,4*Z*)-3,9-dihydroxygermacra-4,10(14),11(13)-trien-12,6-olide.

Artemyrianosin H (**8**) was deduced to have a molecular formula of C_15_H_22_O_4_ with five indices of hydrogen deficiency by its HRESIMS at *m/z* 267.1585 [M + H]^+^ (calcd for C_15_H_23_O_4_, 267.1591). The similarity of ^1^H and ^13^C NMR data (Tables 2 and 3) of **8** and **4** implied structurally closely related, but the major differences were that the oxygenated methine at C-3 and terminal double bond between C-4 and C-15 in compound **4** were absent, meanwhile, a trisubstituent double bond [*δ*_H_ 5.27 (1H, d, *J* = 9.7 Hz, H-3); *δ*_C_ 132.5 (C-3) and 133.5 (C-4)], a singlet methyl [*δ*_H_ 1.74 (3H, s, H-15); *δ*_C_ 20.8 (C-15)], and an oxygenated methine [*δ*_H_ 4.54 (1H, td, *J* = 10.3, 6.0 Hz, H-2); *δ*_C_ 70.8 (C-2)] were appeared in **8**. The spin coupling of H_2_-1/H-2/H-3 in the ^1^H-^1^H COSY spectrum, together with the HMBC correlations from H_3_-15 to C-3/C-4/C-5 and from H_2_-5 to C-3/C-4/C-6/C-7/C-15 verified the above inference. In the ROESY spectrum (Fig. 3), the cross-peak of H-2/H-7 indicated that H-2 and H-7 were *β*-oriented, while the cross-peaks of H-6/H_3_-13 and H-6/H-9 supported their *α*-orientation. In addition, the correlation of H-2/H_3_-15 was clearly observed in the ROESY spectrum, indicating *Δ*^3^-double bond was *E*-configuration. Ultimately, the structure of compound **8** was elucidated as (2*R*,6*S*,7*R*,9*S*,11*S,*3*E*)-2,9-dihydroxygermacra-3,10(14)-dien-12,6-olide by a single-crystal X-ray diffraction experiment with Cu K*α* radiation (Fig. 4).

Artemyrianosin I (**9)** shared the same molecular formula C_15_H_22_O_4_ with **8** according to the HRESIMS at *m/z* 267.1576 [M + H]^+^ (calcd for C_15_H_23_O_4_, 267.1591). Its ^1^H and ^13^C NMR data (Tables 2 and 3) were similar to those of **8**, and detailed interpretation of the ^1^H-^1^H COSY and HMBC spectra revealed the same planar structures. The relative configuration was established through the ROESY cross-peaks of H-2/H-7, H-7/H-13, and H-6/H-9. Likely, the ROESY correlation of H-2/H_3_-15 implied *Δ*^3^-double bond was *E*-configuration. Subsequently, the structure of compound **9** was assigned as (2*R*,6*S*,7*R*,9*S*,11*R,*3*E*)-2,9-dihydroxygermacra-3,10 (14)-dien-12,6-olide by Cu K*α* radiation X-ray crystallographic analysis (Fig. 4).

Artemyrianosin J (**10**) had a molecular formula of C_15_H_20_O_4_ as defined by the HRESIMS at *m/z* 265.1423 [M + H]^+^, which indicated two hydrogen atoms less than compound **9**. The ^1^H and ^13^C NMR data of compound **10** were closely similar to those of **9**, and the main differences were that a doublet methyl at C-13 and a methine at C-11 in compound **9** were replaced by an pair of exocyclic double bond between C-11 and C-13 [*δ*_C_ 141.8 (C-11), 124.7 (C-13); *δ*_H_ 6.24 (1H, d, *J* = 2.8 Hz, H-13a), 5.98 (1H, d, *J* = 2.8 Hz, H-13b)] in compound **10**. This deduction was verified by the spin coupling of H-7/H-11/H_2_-13 in ^1^H-^1^H COSY spectrum and the correlations from H_2_-13 to C-7/C-11/C-12 in the HMBC spectrum. In the ROESY spectrum, the correlation of H-9 with H-2/H-6/H-7 allowed these *β*-orientation. The additional ROESY correlation of H-2/H_3_-15 implied *Δ*^3^-double bond was *E*-configuration. Therefore, the structure of compound **10** was assigned as (2*R*,6*R*,7*R*,9*R,*3*E*)-2,9-dihydroxygermacra-3,10(14),11(13)-trien-12,6-olide based on the similar experimental and calculated ECD curves (Fig. 5).

**Fig. 6:**
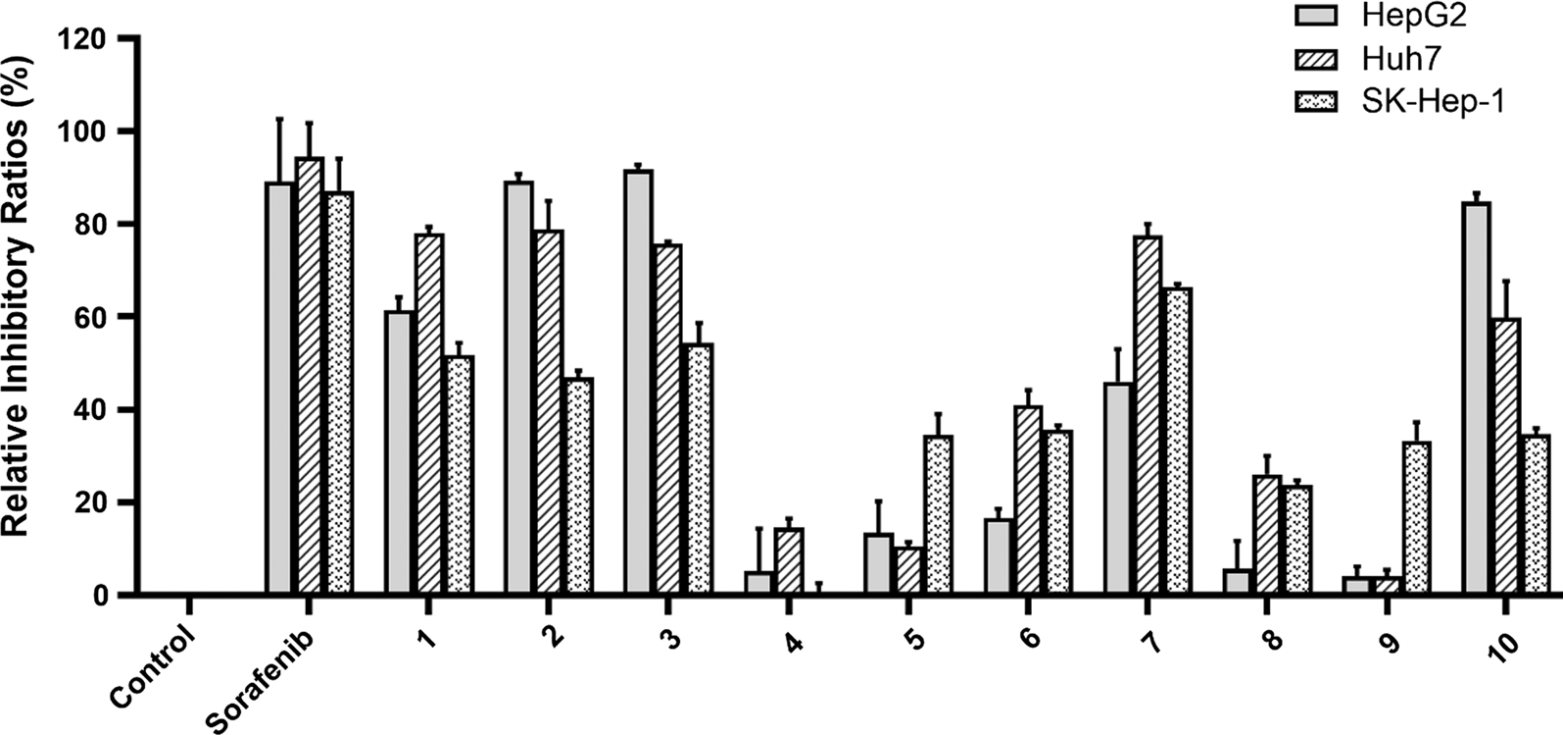
Inhibitory ratios of compounds **1**–**10** at 100 μM

The cytotoxicity of all isolates against three human hepatoma cell lines (HepG2, Huh7, and SK-Hep-1) was evaluated at the concentration of 100 μM with sorafenib as the positive control. As shown in Fig. 6, compounds **1**‒**3**, **7**, and **10** containing an *α*-*exo*-methylene *γ*-butyrolactone group showed activity on HepG2, Huh7, and SK-Hep-1 with inhibitory ratios higher than 50%. The dose–response curves of the active compounds were further investigated to yield their respective IC_50_ values. As shown in Table 4, compounds **1**‒**3** exhibited cytotoxicity against HepG2 cells with IC_50_ values of 43.7‒46.5 μM, while compounds **7** and **10** showed weaker cytotoxicity (IC_50_: 55.1 and 66.1 μM). Meanwhile, compounds **1**‒**3** and **7** also displayed cytotoxicity against Huh7 cells with IC_50_ values ranging from 44.3 to 48.9 μM, but compound **10** showed weaker cytotoxicity with an IC_50_ value of 71.0 μM. For SK-Hep-1 cells, only compound **3** manifested cytotoxicity with an IC_50_ value of 44.9 μM, while other compounds (**1**, **2**, **7** and **10**) showed weaker cytotoxicity with IC_50_ values of 71.7‒89.3 μM. Significantly, compound **3** was the most active one with IC_50_ values of 43.7 (HepG2), 47.9 (Huh7), and 44.9 (SK-Hep-1) μM, respectively.

**Table 4:** Cytotoxicity of compounds **1**–**3**, **7**, and **10**

No	IC_50_ (μM)^a^		
	HepG2	Huh7	SK-Hep-1
**1**	46.5 ± 0.9	44.3 ± 6.0	89.3 ± 0.7
**2**	45.9 ± 3.2	48.9 ± 4.0	71.7 ± 0.4
**3**	43.7 ± 2.8	47.9 ± 4.6	44.9 ± 2.8
**7**	55.1 ± 2.5	44.9 ± 3.0	87.0 ± 1.7
**10**	66.1 ± 4.9	71.0 ± 2.8	74.8 ± 1.2
Sorafenib^b^	13.5 ± 2.2	20.7 ± 1.1	11.8 ± 0.6

^a^Data were expressed as means ± SD (n = 3)

^b^Sorafenib was used as a positive control

## Conclusion

In this study, 10 new germacrane-type sesquiterpenoids (**1**–**10**) were isolated and identified from *A. myriantha*. Their structures were elucidated by extensive analyses of spectral data, X-ray analyses, and ECD spectra. Compounds **1**‒**3** showed cytotoxicity against HepG2 cells with IC_50_ values of 43.7‒46.5 μM; compounds **1**‒**3** and **7** had cytotoxicity against Huh7 cell lines with IC_50_ values ranging from 44.3 to 48.9 μM; only compound **3** exhibited cytotoxicity against SK-Hep-1 cells with IC_50_ value of 44.9 μM. Interestingly, compound **3** displayed cytotoxicity against three human hepatoma cell lines with IC_50_ values of 43.7 (HepG2), 47.9 (Huh7), and 44.9 (SK-Hep-1) μM, respectively. This investigation provided valuable information for the understanding of antihepatoma parts of *A. myriantha* and germacrane-type sesquiterpenoids as the active constituents.

## Materials and methods

General experimental procedures, the ECD calculation, and cytotoxicity assays were provided in Additional file 1.

### Plant materials

*Artemisia myriantha* was collected from Lijiang, Yunnan province, China in September 2018, and identified by Dr. Zhuo Zhou (Kunming Institute of Botany, Chinese Academy of Sciences). A voucher specimen (No. 201809AM) was deposited in the laboratory of Antivirus and Natural Medicinal Chemistry, Kunming Institute of Botany, Chinese Academy of Sciences, Kunming, China.

### Extraction and isolation

In connection with our previous paper [25], Fr. E (165 g) was chromatographed on a silica gel column (1.6 kg, 10 × 90 cm, MeOH–CHCl_3_, 2:98–10:90, *v/v*) to obtain fractions E1–E4 (25, 30, 46 and 55 g). Fraction E2 was subjected to MCI gel CHP 20P column (490 g, 5.0 × 50 cm) and eluted with a H_2_O–MeOH gradient (70:30, 50:50, 30:70, 0:100) to yield four subfractions E2.1–E2.4. Fraction E2.2 (18 g) was applied to Si CC (200 g, 5.0 cm × 25 cm) and eluted with an EtOAc-CHCl_3_ gradient (10:90, 20:80 and 30:70) to produce four subfractions E2.2.1–E2.2.4. The obtained fraction E.2.2.2 (2.2 g) was separated by preparative HPLC (H_2_O–MeCN, 76:24, 10.0 mL/min) to afford three fractions (E2.2.2.1–E2.2.2.3). Fraction E2.2.2.1 (218 mg) was purified by semipreparative HPLC (H_2_O–MeOH, 72:28, 3.0 mL/min) to yield compounds **1** (22 mg, *t*_R_ = 28.3 min), **2** (13 mg, *t*_R_ = 26.5 min), and **3** (25 mg, *t*_R_ = 30.6 min). Fraction E2.2.3 (1.9 g) was isolated by repeated silica gel CC (50 g, 2.5 × 20 cm, EtOAC–CHCl_3_, 10:90–30:70) and semipreparative HPLC (H_2_O–MeCN, 75:25) to get compounds **4** (14 mg, *t*_R_ = 27.8 min) and **8** (8 mg, *t*_R_ = 29.3 min). Fraction E3 (46 g) was fractioned by MPLC on an MCI gel CHP 20P column (490 g, 5 cm × 50 cm) with a gradient solvent system of H_2_O–MeOH (80:20, 60:40, 40:60, 0:100) to provide four subfractions E3.1–E3.4 (16, 6.5, 8.9, and 18 g). Fraction E3.2 (6.5 g) was fractionated with Si CC (80 g, 3.5 × 35 cm) using EtOAc–CHCl_3_ (10:90–30:70) to afford three subfractions E3.2.1 − E3.2.3 (2.5, 1.6 and 1.8 g). The obtained fraction E3.2.2 (1.6 g) was further isolated by Sephadex LH-20 CC (120 g, 2.5 × 150 cm, MeOH) and semipreparative HPLC (H_2_O − MeCN, 82:18, 3.0 mL/min) to provide compounds **7** (18 mg, *t*_R_ = 24.3 min), **9** (6 mg, *t*_R_ = 21.2 min), and **10** (5 mg, *t*_R_ = 22.8 min). Fraction E3.3 (8.9 g) was chromatographed over a silica gel column (110 g, 4.5 × 20 cm) eluted with an EtOAc − CHCl_3_ gradient (10:90, 20:80 and 30:70) to give fractions E3.3.1–E3.3.4. Fraction E3.3.2 (2.4 g) was conducted on preparative HPLC (H_2_O–MeCN, 82:18, 10 mL/min) and semipreparative HPLC (H_2_O − MeOH, 72:28, 3.0 mL/min) to yield compounds **5** (28 mg, *t*_R_ = 35.3 min) and **6** (15 mg, *t*_R_ = 32.8 min).

### Spectroscopic data of compounds 1–10

#### Artemyrianosin A (1)

Colorless monoclinic crystals (MeOH-CHCl_3_, 95:5); mp 153.8–155.2 ℃; [*α*]25 D + 12.5 (*c* 0.11, MeOH); ECD (MeOH) *λ*max (Δ*ε*) 199 (− 4.0), 218 (+ 3.4), 250 (+ 1.3) nm; IR v_max_ 3414, 1757, 1643, 1570, 1457, 1414, 1384, 1276, 1155, 1016, 994 cm^‒1^; ^1^H and ^13^C NMR data see Tables 1 and 2; (+)-HRESIMS m/*z* 287.1254 [M + Na]^+^ (calcd for C_15_H_20_O_4_Na, 287.1254).

#### Artemyrianosin B (2)

White amorphous powder; [*α*]_26_^D^ + 37.9 (*c* 0.11, MeOH); ECD (MeOH) *λ*max (Δ*ε*) 196 (− 0.8), 220 (+ 4.6) nm; IR *v*_max_ 3430, 1742, 1644, 1449, 1384, 1276, 1160, 1001, 908 cm^‒1^; ^1^H and ^13^C NMR data see Tables 1 and 2; (+)-HRESIMS *m/z* 265.1427 [M + H]^+^ (calcd for C_15_H_21_O_4_, 265.1434).

#### Artemyrianosin C (3)

White amorphous powder; [*α*]_25_^D^ + 31.2 (*c* 0.11, MeOH); ECD (MeOH) *λ*max (Δ*ε*) 197 (+ 1.4), 212 (+ 3.4) nm; IR *v*_max_ 3424, 3388, 1767, 1648, 1632, 1426, 1384, 1323, 1286, 1116, 989 cm^‒1^; ^1^H and ^13^C NMR data see Tables 1 and 2; (+)-HRESIMS *m/z* 265.1431 [M + H]^+^ (calcd for C_15_H_21_O_4_, 265.1434).

#### Artemyrianosin D (4)

White amorphous powder; [*α*]_24_^D^ + 48.4 (*c* 0.11, MeOH); ECD (MeOH) *λ*max (Δ*ε*) 201 (− 3.6), 244 (+ 0.2) nm;IR *v*_max_ 3427, 1759, 1634, 1459, 1384, 1346, 1186, 1039, 991, 908 cm^‒1^; ^1^H and ^13^C NMR data see Tables 1 and 2; (+)-HRESIMS *m/z* 267.1579 [M + H]^+^ (calcd for C_15_H_23_O_4_, 267.1591).

#### Artemyrianosin E (5)

Colorless oil; [*α*]_25_^D^ − 3.9 (*c* 0.10, MeOH); ECD (MeOH) *λ*max (Δ*ε*) 197 (− 5.7), 224 (+ 1.0) nm; IR *v*_*max*_ 3428, 1718, 1631, 1439, 1384, 1245, 1150, 1020, 909 cm^‒1^; ^1^H and ^13^C NMR data see Tables 1 and 2; (+)-HRESIMS *m/z* 339.1792 [M + H]^+^ (calcd for C_18_H_27_O_6_, 339.1802).

#### Artemyrianosin F (6)

Colorless oil; [*α*]_25_^D^ − 17.6 (*c* 0.12, MeOH); ECD (MeOH) *λ*max (Δ*ε*) 202 (− 10.3), 224 (+ 1.7) nm; IR *v*_*max*_ 3423, 1719, 1629, 1439, 1382, 1246, 1152, 1027, 908 cm^‒1^; ^1^H and ^13^C NMR data see Tables 2 and 3; (+)-HRESIMS *m/z* 339.1785 [M + H]^+^ (calcd for C_18_H_27_O_6_, 339.1802).

#### Artemyrianosin G (7)

Colorless monoclinic crystals (MeOH-CHCl_3_, 95:5); mp 154.2–156.1 ℃; [*α*]_25_^D^ + 66.8 (*c* 0.14, MeOH); ECD (MeOH) *λ*max (Δ*ε*) 203 (+ 8.5), 220 (+ 3.2) nm; IR *v*_max_ 3467, 3435, 1749, 1664, 1643, 1632, 1445, 1410, 1381, 1314, 1263, 1129, 1027 cm^‒1^; ^1^H and ^13^C NMR data see Tables 2 and 3; (+)-HRESIMS *m/z* 265.1423 [M + H]^+^ (calcd for C_15_H_21_O_4_, 265.1434).

#### Artemyrianosin H (8)

Colorless monoclinic crystals (MeOH-CHCl_3_, 95:5); mp 151.2–153.1 ℃; [*α*]_24_^D^ –83.8 (*c* 0.13, MeOH); ECD (MeOH) *λ*max (Δ*ε*) 202 (–27.1), 227 (+ 0.5) nm; IR *v*_max_ 3500, 3367, 1751, 1667, 1650, 1452, 1384, 1195, 1184, 1021, 1010 cm^‒1^; ^1^H and ^13^C NMR data see Tables 2 and 3; (+)-HRESIMS *m/z* 267.1585 [M + H]^+^ (calcd for C_15_H_23_O_4_, 267.1591).

#### Artemyrianosin I (9)

Colorless tetragonal crystals (MeOH-CHCl_3_, 95:5); mp 150.8–152.3 ℃; [*α*]_26_^D^ –2.6 (*c* 0.12, MeOH); ECD (MeOH) *λ*max (Δ*ε*) 202 (–3.9), 229 (+ 0.2) nm; IR *v*_max_ 3391, 3308, 1751, 1632, 1564, 1384, 1064 cm^‒1^; ^1^H and ^13^C NMR data see Tables 2 and 3; (+)-HRESIMS *m/z* 267.1576 [M + H]^+^ (calcd for C_15_H_23_O_4_, 267.1591).

#### Artemyrianosin J (10)

White amorphous powder; [*α*]_26_^D^ –11.4 (*c* 0.13, MeOH); ECD (MeOH) *λ*max (Δ*ε*) 201 (+ 4.0), 222 (+ 0.8) nm; IR *v*_max_ 3391, 1754, 1631, 1594, 1567, 1384, 1073 cm^‒1^; ^1^H and ^13^C NMR data see Tables 2 and 3; (+)-HRESIMS *m/z* 265.1423 [M + H]^+^ (calcd for C_15_H_21_O_4_, 265.1434).

### X-ray crystallographic analysis of compounds 1 and 7‒9

Compounds **1** and **7**‒**9** were afforded by recrystallization in a mixture of MeOH–CHCl_3_ (95:5). X-ray diffraction analyses were performed on a Bruker D8 QUEST instrument using Cu K*α* radiation and the intensity data were collected at 100 (2) K. The crystal structures were solved by using SHELXS-97 and difference Fourier techniques, and refinements were performed through the program and refined by full-matrix least-squares calculations on F^2^. All non-hydrogen atoms were anisotropically refined, and the positions of hydrogens bonded to carbons were initially determined through geometry and refined using a riding model. The crystallographic data for compounds **1** and **7**‒**9** in standard CIF format were deposited in the Cambridge Crystallographic Data Centre. The data can be accessed free of charge at http://www.ccdc.cam.ac.uk/.

*Crystal data for compound ****1***: C_15_H_20_O_4_, *M* = 264.31, *a* = 8.1236 (3) Å, *b* = 10.9775 (5) Å, *c* = 15.0127 (6) Å, *α* = 90°, *β* = 95.6520 (10)°, *γ* = 90°, *V* = 1332.28 (9) Å^3^, *T* = 100. (2) K, space group *P*1211, *Z* = 4, *μ* (Cu Kα) = 0.774 mm^−1^, 33,394 measured reflections, 5199 independent reflections (*R*_*int*_ = 0.0763). The final *R*_*1*_ values were 0.0397 [*I* > 2*σ* (*I*)]. The final *wR* (*F*^2^) values were 0.1190 [*I* > 2*σ* (*I*)]. The final *R*_*1*_ values were 0.0447 (all data). The final *wR* (*F*^2^) values were 0.1207 (all data). The goodness of fit on *F*^2^ was 1.115. Flack parameter = 0.11 (6). CCDC 2,142,473.

*Crystal data for compound ****7***: C_15_H_20_O_4_, *M* = 264.31, *a* = 5.6882 (2) Å, *b* = 15.5718 (4) Å, *c* = 7.6450 (2) Å, *α* = 90°, *β* = 93.2180 (10)°, *γ* = 90°, *V* = 676.09 (3) Å^3^, *T* = 100. (2) K, space group *P*1211, *Z* = 2, *μ* (Cu Kα) = 0.762 mm^−1^, 14,454 measured reflections, 2570 independent reflections (*R*_*int*_ = 0.0451). The final *R*_*1*_ values were 0.0379 [*I* > 2*σ* (*I*)]. The final *wR* (*F*^2^) values were 0.0984 [*I* > 2*σ* (*I*)]. The final *R*_*1*_ values were 0.0379 (all data). The final *wR* (*F*^2^) values were 0.0985 (all data). The goodness of fit on *F*^2^ was 1.070. Flack parameter = 0.04 (8). CCDC 2,142,472.

*Crystal data for compound ****8***: C_15_H_22_O_4_·2 (H_2_O), *M* = 302.36, *a* = 10.1051 (5) Å, *b* = 5.9207 (3) Å, *c* = 13.4788 (6) Å, *α* = 90°, *β* = 96.4640 (10)°, *γ* = 90°, *V* = 801.30 (7) Å^3^, *T* = 100. (2) K, space group *P*1211, *Z* = 2, *μ* (Cu Kα) = 0.796 mm^−1^, 13,564 measured reflections, 3137 independent reflections (*R*_*int*_ = 0.0336). The final *R*_*1*_ values were 0.0292 [*I* > 2*σ* (*I*)]. The final *wR* (*F*^2^) values were 0.0752 [*I* > 2*σ* (*I*)]. The final *R*_*1*_ values were 0.0294 (all data). The final *wR* (*F*^2^) values were 0.0755 (all data). The goodness of fit on *F*^2^ was 1.081. Flack parameter = 0.06 (4). CCDC 2,142,471.

*Crystal data for compound ****9***: C_15_H_22_O_4_, *M* = 266.32, *a* = 8.2909 (4) Å, *b* = 8.2909 (4) Å, *c* = 40.8360 (19) Å, *α* = 90°, *β* = 90°, *γ* = 90°, *V* = 2807.0 (3) Å^3^, *T* = 100. (2) K, space group *P*41212, *Z* = 8, *μ* (Cu Kα) = 0.735 mm^−1^, 44,745 measured reflections, 2766 independent reflections (*R*_*int*_ = 0.0520). The final *R*_*1*_ values were 0.0273 [*I* > 2*σ* (*I*)]. The final *wR* (*F*^2^) values were 0.0705 [*I* > 2*σ* (*I*)]. The final *R*_*1*_ values were 0.0273 (all data). The final *wR* (*F*^2^) values were 0.0706 (all data). The goodness of fit on *F*^2^ was 1.091. Flack parameter = 0.05 (2). CCDC 2,142,474.

## Supplementary Information

**Additional file 1.** Supporting Information.
