# Supplementary material for: Artemyrianosins A–J, cytotoxic germacrane-type sesquiterpene lactones from Artemisia myriantha
Source: Nat Prod Bioprospect. 2022 May 2;12(1):16. doi: 10.1007/s13659-022-00340-5 (PMC9058048; doi:10.1007/s13659-022-00340-5)
Supplement: Supplementary file 1 — Additional file 1. Supporting Information. [file 13659_2022_340_MOESM1_ESM.docx]

**Supporting Information**

**Artemyrianosins A–J, Cytotoxic** **Germacrane-Type Sesquiterpene Lactones from *Artemisia*** ***myriantha***

Xin Zhang^a,b^, Yun-Bao Ma^a^, Xiao-Feng He^a^, Tian-Ze Li^a^, Chang-An Geng^a^, Li-Hua Su^a^, Shuang Tang^a^, Zhen Gao^a,b^, Ji-Jun Chen^a,b*^

^a^ State Key Laboratory of Phytochemistry and Plant Resources in West China, Yunnan Key Laboratory of Natural Medicinal Chemistry, Kunming Institute of Botany, Chinese Academy of Sciences, Kunming 650201, People's Republic of China

^b^ University of Chinese Academy of Sciences, Beijing 100049, People's Republic of China

*Corresponding author. Prof. Dr. Ji-Jun Chen, State Key Laboratory of Phytochemistry and Plant Resources in West China, Kunming Institute of Botany, Chinese Academy of Sciences, 132# Lanhei Road, Kunming 650201, Yunnan, People's Republic of China

Phone: + 86-871-65223265, Fax: + 86-871-65227197, E-mail address: chenjj@mail.kib.ac.cn

**Contents**

[General experimental procedures 3](#_Toc97303172)

[Cytotoxicity assay 3](#_Toc97303173)

[ECD calculations 3](#_Toc97303174)

[1. NMR, MS, IR, [*a*]_D_ and CD spectra of compound 1 4](#_Toc97303175)

[2. NMR, MS, IR, [*a*]_D_ and CD spectra of compound 2 14](#_Toc97303176)

[3. NMR, MS, IR, [*a*]_D_ and CD spectra of compound 3 24](#_Toc97303177)

[4. NMR, MS, IR, [*a*]_D_ and CD spectra of compound 4 34](#_Toc97303178)

[5. NMR, MS, IR, [*a*]_D_ and CD spectra of compound 5 44](#_Toc97303179)

[6. NMR, MS, IR, [*a*]_D_ and CD spectra of compound 6 54](#_Toc97303180)

[7. NMR, MS, IR, [*a*]_D_ and CD spectra of compound 7 64](#_Toc97303181)

[8. NMR, MS, IR, [*a*]_D_ and CD spectra of compound 8 74](#_Toc97303182)

[9. NMR, MS, IR, [*a*]_D_ and CD spectra of compound 9 84](#_Toc97303183)

[13. NMR, MS, IR, [*a*]_D_ and CD spectra of compound 10 94](#_Toc97303184)

### **General experimental procedures**

Optical rotations were conducted on an Autopol VI automatic polarimeter (Rudolph Research Analytical, Hackettstown, NJ, USA). IR (KBr) spectra were measured on a Nicolet iS10 (Thermo Fisher Scientific, Madison, WI, USA). ECD spectra were obtained on an Applied Photophysics Chirascan apparatus (Applied Photophysics, Surrey, UK). 1D and 2D NMR experiments were carried out using Avance III-500 and III-600 spectrometers (Bruker, Bremerhaven, Germany) with TMS as an internal standard. HRESIMS data were recorded on an LCMS-IT-TOF mass spectrometer (Shimadzu, Kyoto, Japan). Silica gel (200−300 mesh, Linyi Haixiang Co. Ltd., Linyi, China) and Sephadex LH-20 gel (Amersham Biosciences, Uppsala, Sweden) were employed for column chromatography. Thin-layer chromatography (TLC) analyses were performed on silica gel GF_254_ plates (Jiangyou, Chemical Co. Ltd., Yantai, China). MPLC separations were conducted on a Dr-Flash II apparatus (Lisui, Suzhou, China) using an MCI gel CHP 20P column (75−150 *μ*m, Mitsubishi Chemical Corporation, Tokyo, Japan). HPLC puriﬁcation was achieved on an LC-CBM-20 system (Shimadzu, Kyoto, Japan) with an Agilent Eclipse XDB-C_18_ column (5 *μ*m, 9.4 × 250 mm, Agilent Technologies, Santa Clara, USA).

### **Cytotoxicity assay**

The antihepatoma activity of all the isolates were measured by MTT assay on three human hepatocellular carcinoma cell lines (HepG2, Huh7, and SK-Hep-1)^19^. The cells were harvested and seeded in a 96-well plate at a density of 1 × 10^4^ cells per well. After incubating the cells with 5% CO_2_ at 37 °C for 24 h, samples with different concentrations were added to cells and incubated for 48 h. Afterwards, 100 μL of MTT solution (1 mg/mL) was added into each well and co-incubated for 4 h at 37 ℃. Then the solution was removed, and 100 μL of DMSO was added to dissolve the MTT formazan salt. The absorbance was measured using a microplate reader (BIO-RAD, USA) at 490 nm. The inhibitory ratios were calculated as [A_(control)_ – A_(sample)_] / A_(control)_ × 100%, and IC_50_ values were calculated by GraphPad Prism 7 (GraphPad Software, San Diego, CA, USA). All results and data were expressed as mean ± SD at three independent experiments.

### **ECD calculations**

The relative conﬁgurations of compounds **2**–**6**, and **10** were established according to their ROESY experiments and optimized by DFT calculation at the B3lyp/6-31G (d, p) level in the gas phase. To exclude imaginary frequencies, frequency calculations were performed at the same level. ECD calculations were performed using the TDDFT methodology at the b3lyp/6-311+g (d, p) level with the consideration of solvent eﬀects. ECD calculations were performed using the Gaussian 09 program package. The ECD curves were drawn using the Origin Pro 9 program (OriginLab Corporation, Northampton, MA, USA).

1. NMR, MS, IR, [*a*]_D_ and CD spectra of compound 1

Figure S1. ^1^H NMR spectrum (600 MHz) of artemyrianosin A (**1**) in CD_3_OD

Figure S2. ^13^C NMR spectra (150 MHz) of artemyrianosin A (**1**) in CD_3_OD

Figure S3. HSQC spectrum (600 MHz) of artemyrianosin A (**1**) in CD_3_OD

Figure S4. HMBC spectrum (600 MHz) of artemyrianosin A (**1**) in CD_3_OD

Figure S5. ^1^H–^1^H COSY spectrum (600 MHz) of artemyrianosin A (**1**) in CD_3_OD

Figure S6. ROESY spectrum (600 MHz) of artemyrianosin A (**1**) in CD_3_OD


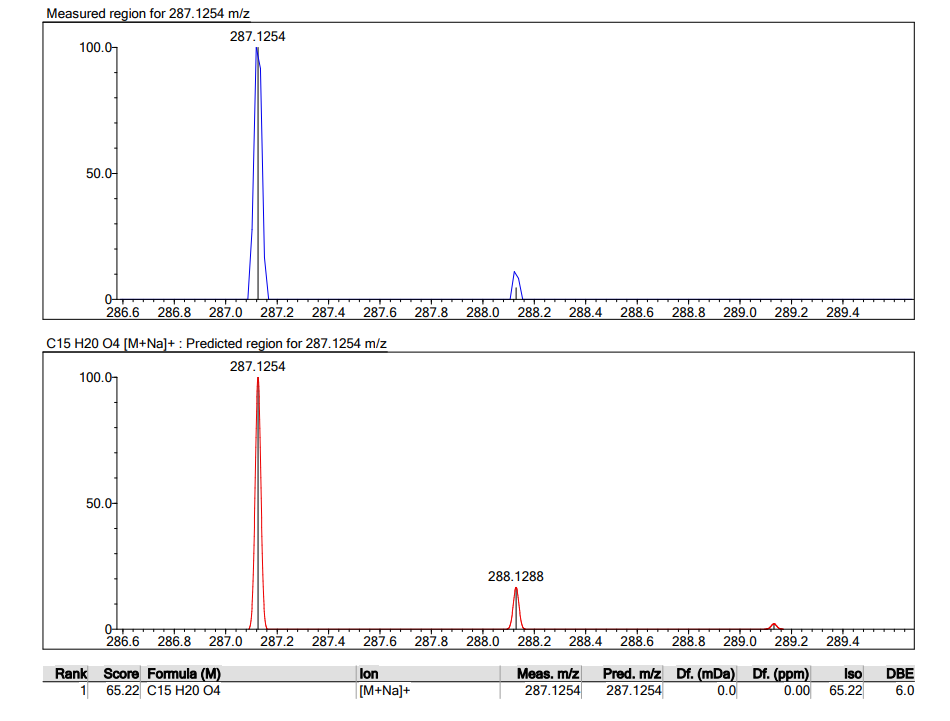


Figure S7. HRESIMS spectrum of artemyrianosin A (**1**)


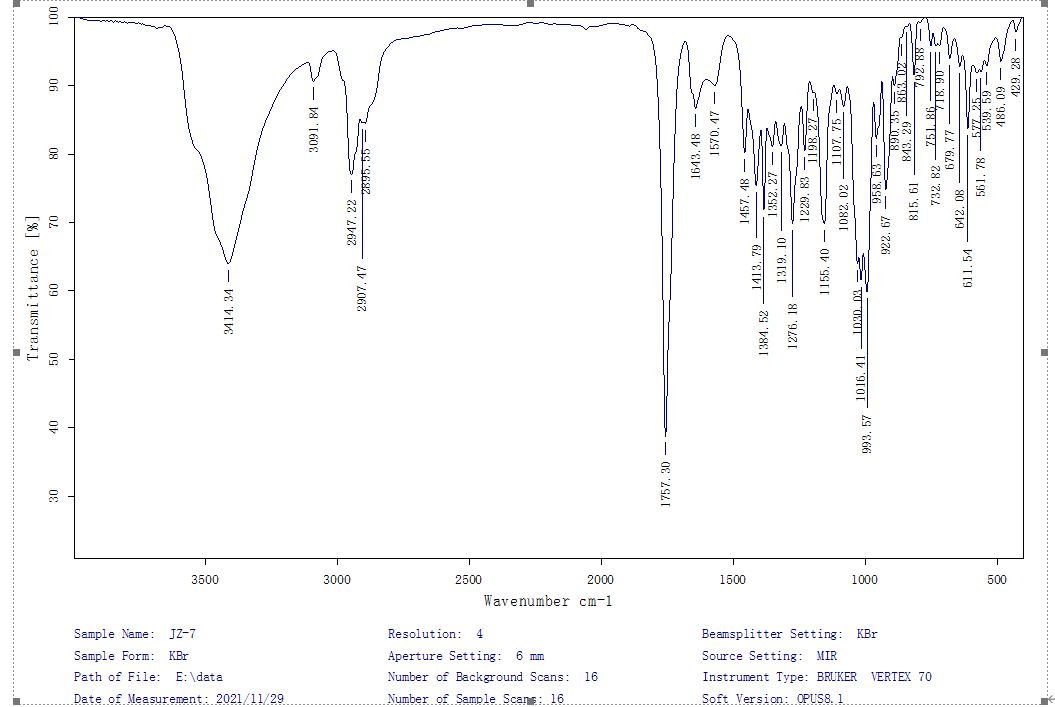


Figure S8. IR spectrum of artemyrianosin A (**1**)


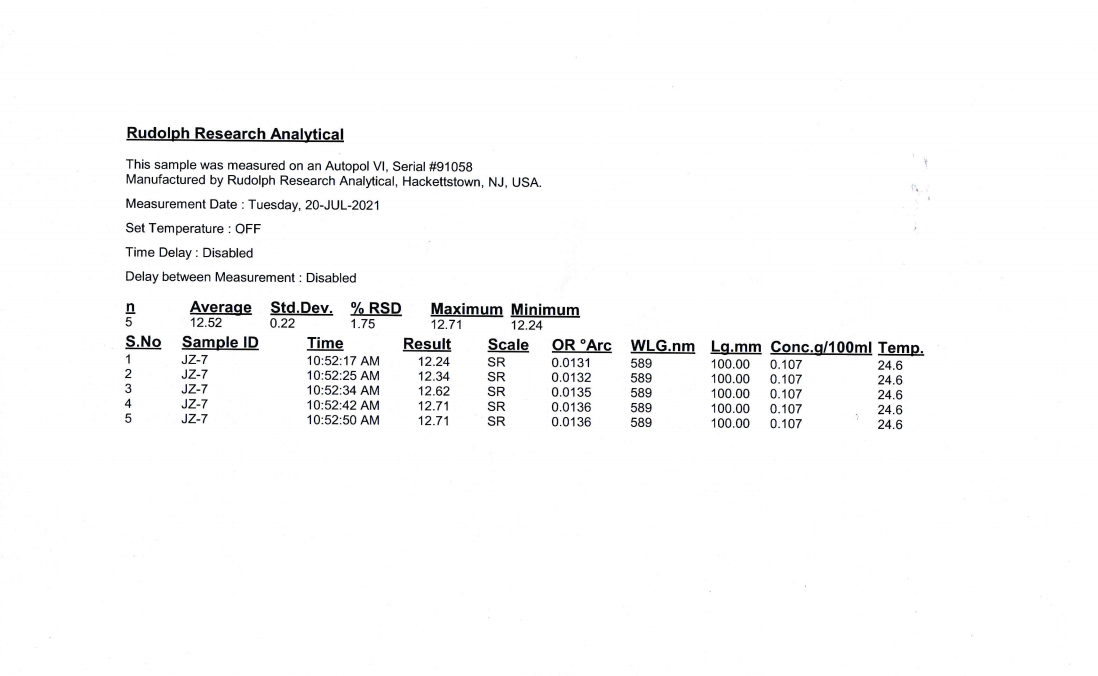


Figure S9. Optical rotation spectrum of artemyrianosin A (**1**)


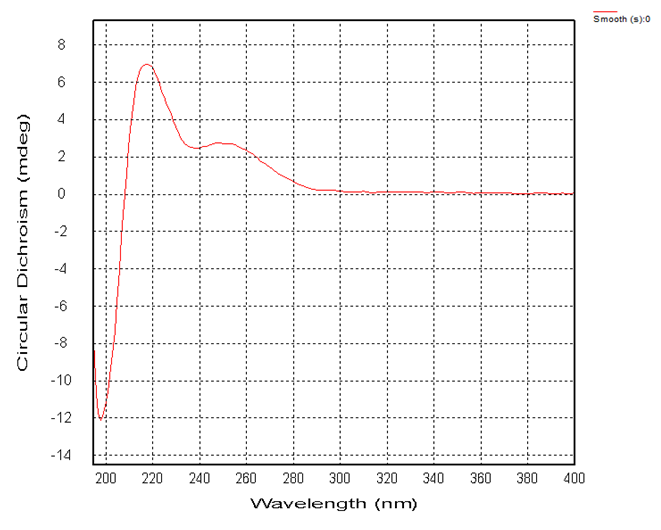


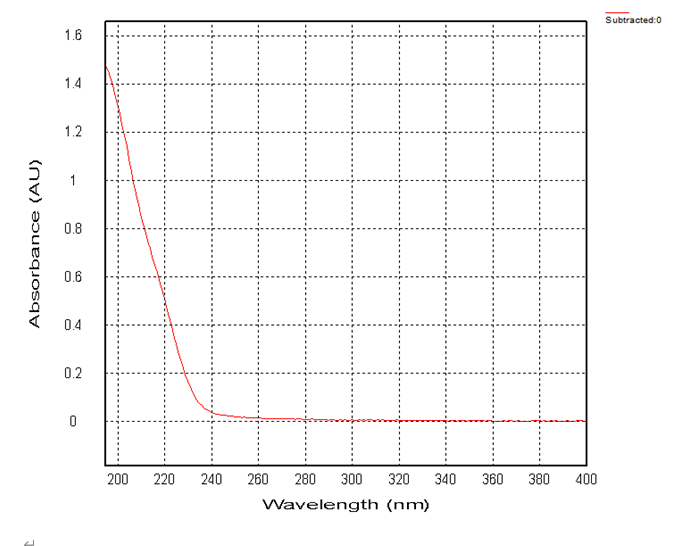


Figure S10. CD (top) and UV (bottom) spectra of artemyrianosin A (**1**)

2. NMR, MS, IR, [*a*]_D_ and CD spectra of compound 2

Figure S11. ^1^H NMR spectrum (600 MHz) of artemyrianosin B (**2**) in CD_3_OD

Figure S12. ^13^C NMR spectrum (150 MHz) of artemyrianosin B (**2**) in CD_3_OD

Figure S13. HSQC spectrum (600 MHz) of artemyrianosin B (**2**) in CD_3_OD

Figure S14. HMBC spectrum (600 MHz) of artemyrianosin B (**2**) in CD_3_OD


Figure S15. ^1^H–^1^H COSY spectrum (600 MHz) of artemyrianosin B (**2**) in CD_3_OD

Figure S16. ROESY spectrum of (600 MHz) artemyrianosin B (**2**) in CD_3_OD


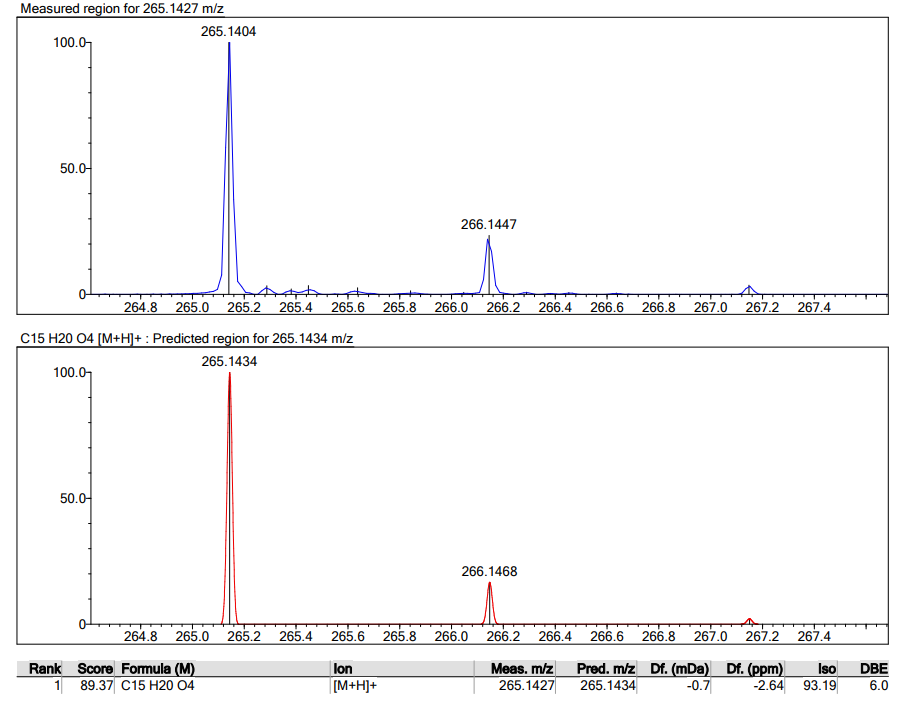


Figure S17. HRESIMS spectrum of artemyrianosin B (**2**)


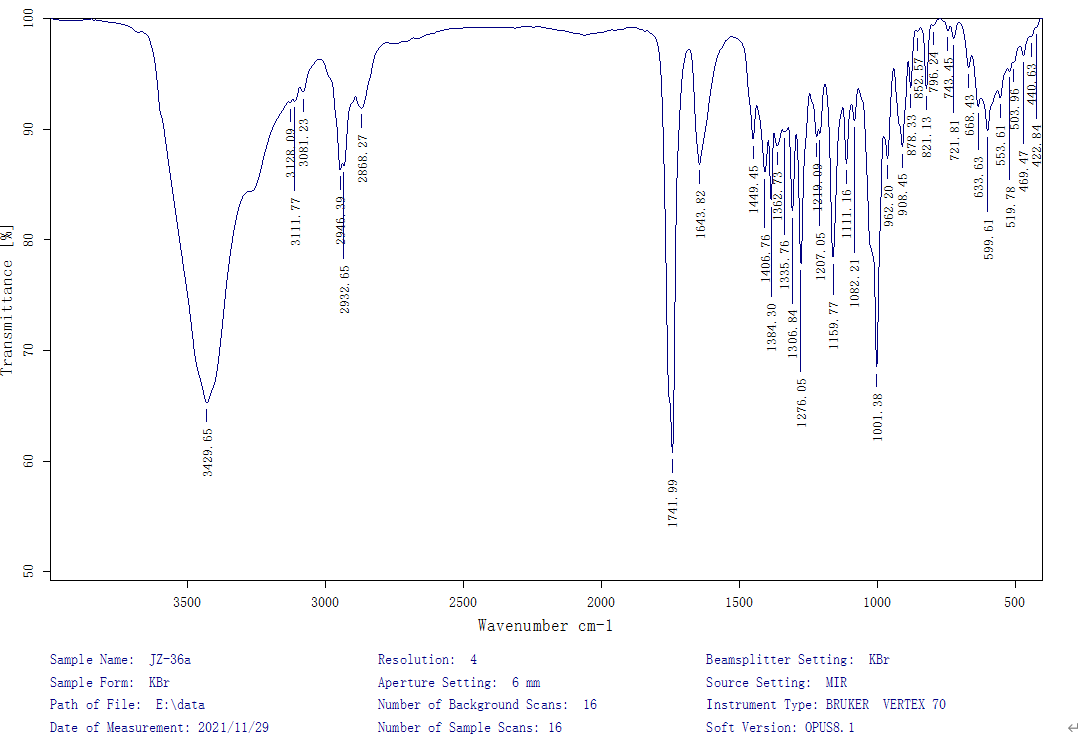


Figure S18. IR spectrum of artemyrianosin B (**2**)


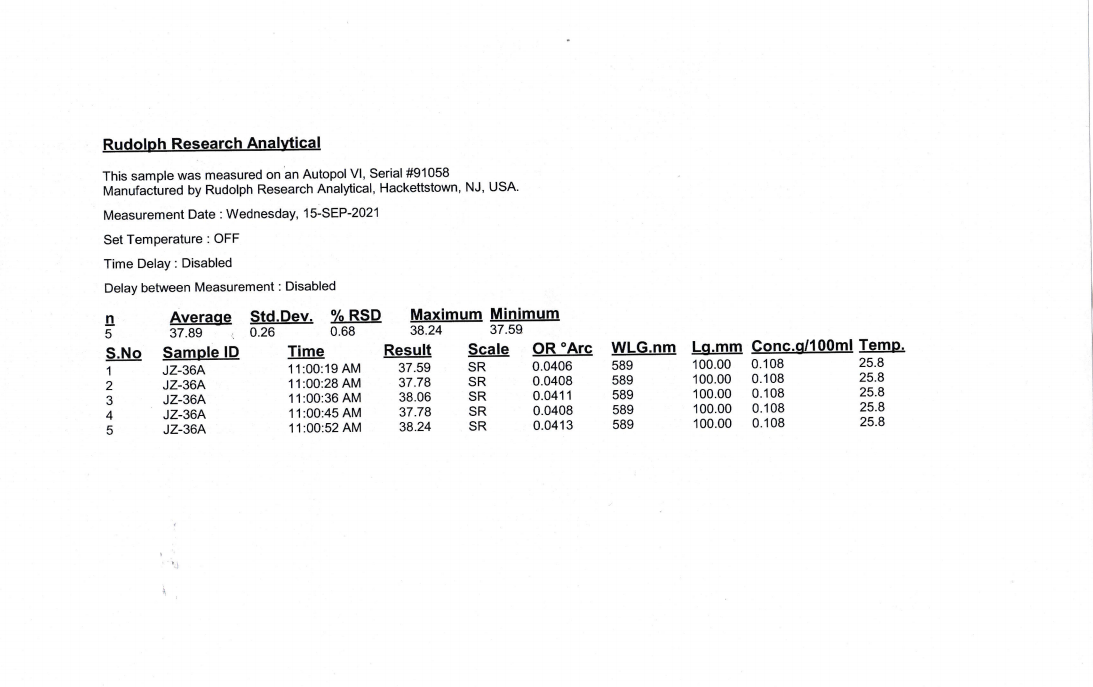


Figure S19. Optical rotation spectrum of artemyrianosin B (**2**)


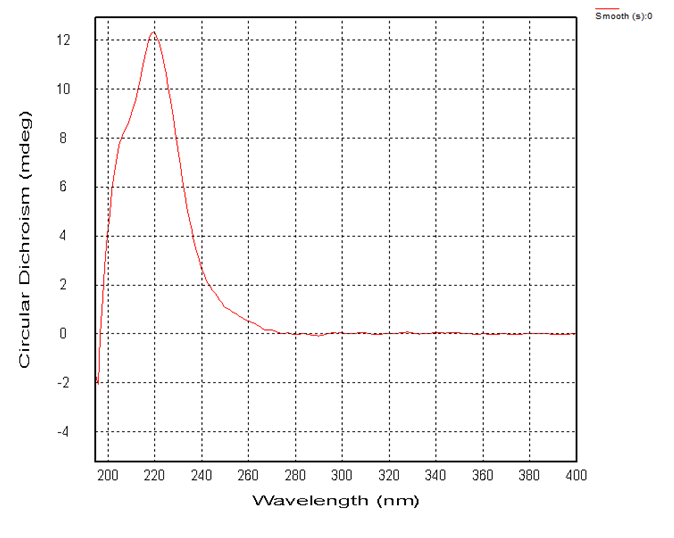


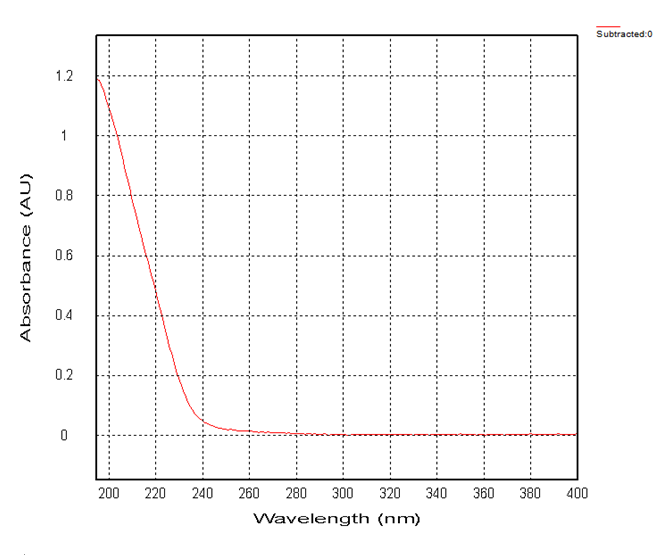


Figure S20. CD (top) and UV (bottom) spectra of artemyrianosin B (**2**)

3. NMR, MS, IR, [*a*]_D_ and CD spectra of compound 3

Figure S21. ^1^H NMR spectrum (600 MHz) of artemyrianosin C (**3**) in CD_3_OD

Figure S22. ^13^C NMR spectrum (150 MHz) of artemyrianosin C (**3**) in CD_3_OD

Figure S23. HSQC spectrum (600 MHz) of artemyrianosin C (**3**) in CD_3_OD

Figure S24. HMBC spectrum (600 MHz) of artemyrianosin C (**3**) in CD_3_OD

Figure S25. ^1^H–^1^H COSY spectrum (600 MHz) of artemyrianosin C (**3**) in CD_3_OD

Figure S26. ROESY spectrum (600 MHz) of artemyrianosin C (**3**) in CD_3_OD


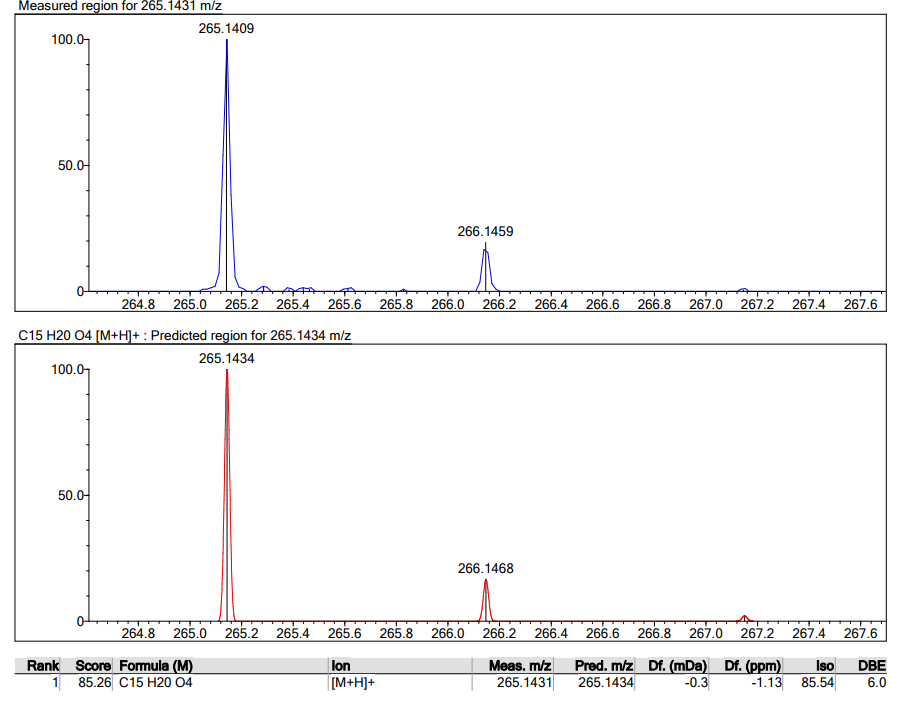


Figure S27. HRESIMS spectrum of artemyrianosin C (**3**)


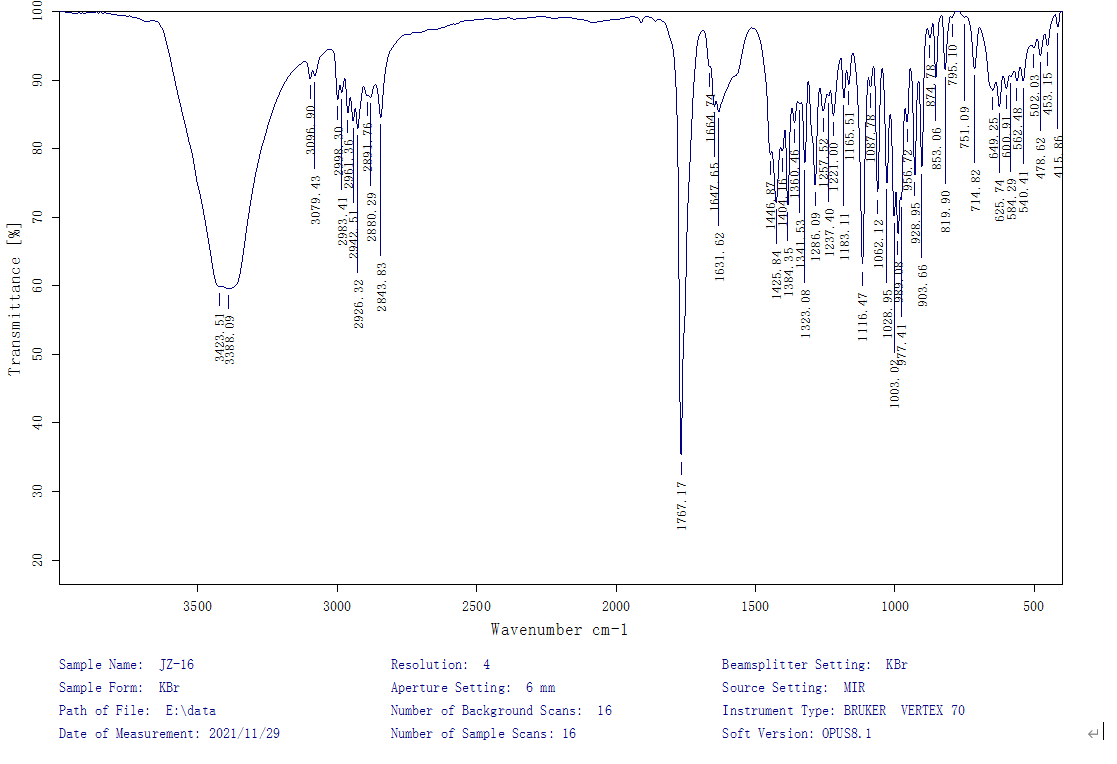


Figure S28. IR spectrum of artemyrianosin C (**3**)


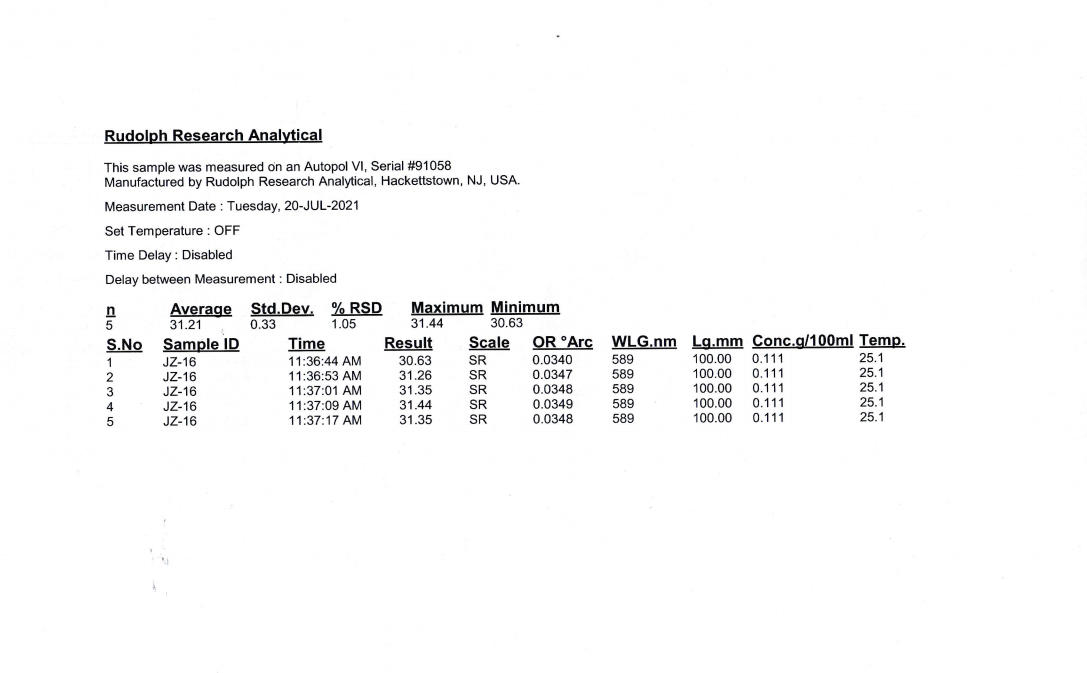


Figure S29. Optical rotation spectrum of artemyrianosin C (**3**)


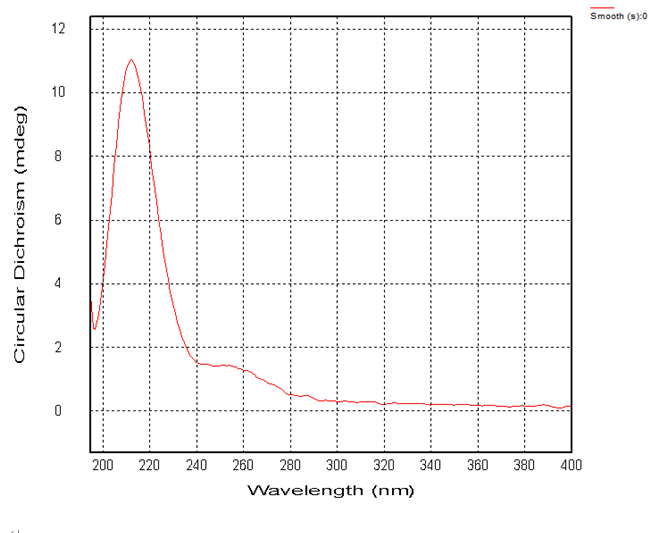

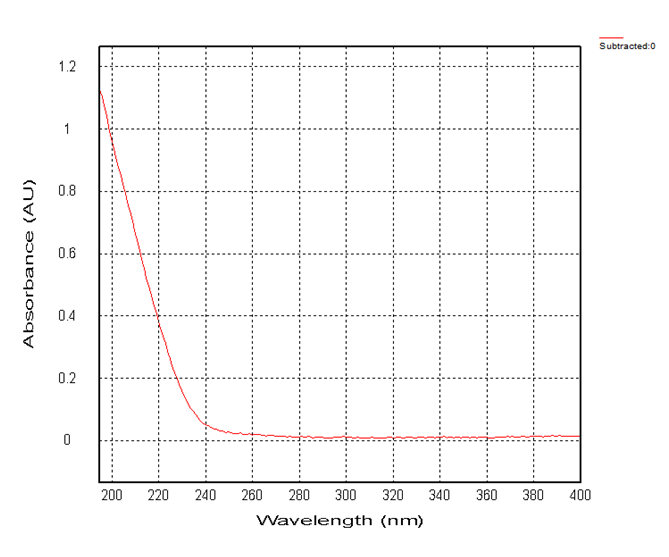


Figure S30. CD (top) and UV (bottom) spectra of artemyrianosin C (**3**)

4. NMR, MS, IR, [*a*]_D_ and CD spectra of compound 4

Figure S31. ^1^H NMR spectrum (600 MHz) of artemyrianosin D (**4**) in CD_3_OD

Figure S32. ^13^C NMR spectrum (150 MHz) of artemyrianosin D (**4**) in CD_3_OD

Figure S33. HSQC spectrum (600 MHz) of artemyrianosin D (**4**) in CD_3_OD

Figure S34. HMBC spectrum (600 MHz) of artemyrianosin D (**4**) in CD_3_OD

Figure S35. ^1^H–^1^H COSY spectrum (600 MHz) of artemyrianosin D (**4**) in CD_3_OD

Figure S36. ROESY spectrum (600 MHz) of artemyrianosin D (**4**) in CD_3_OD


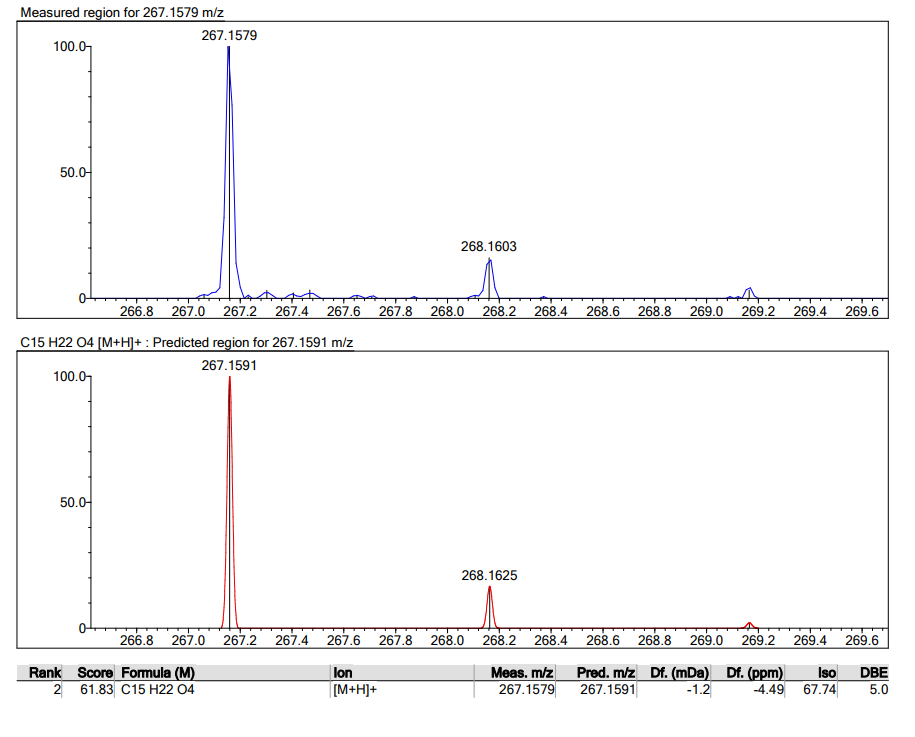


Figure S37. HRESIMS spectrum of artemyrianosin D (**4**)


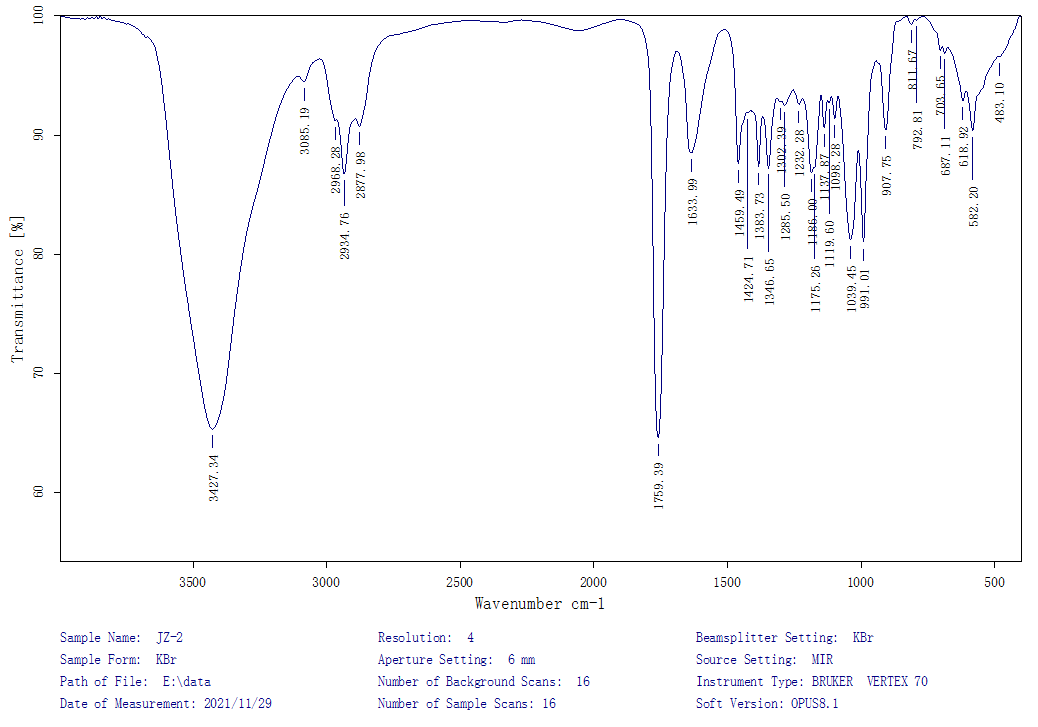


Figure S38. IR spectrum of artemyrianosin D (**4**)


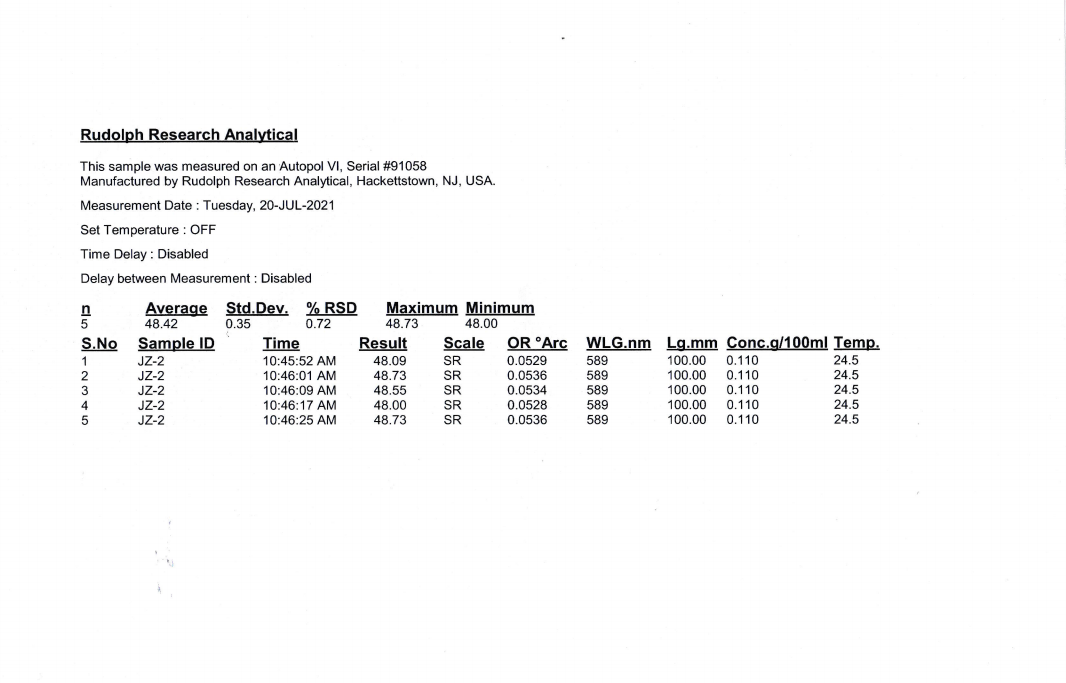


Figure S39. Optical rotation spectrum of artemyrianosin D (**4**)


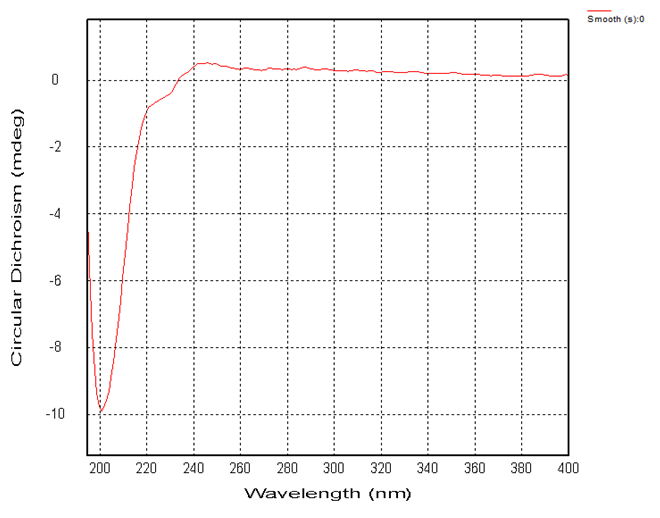

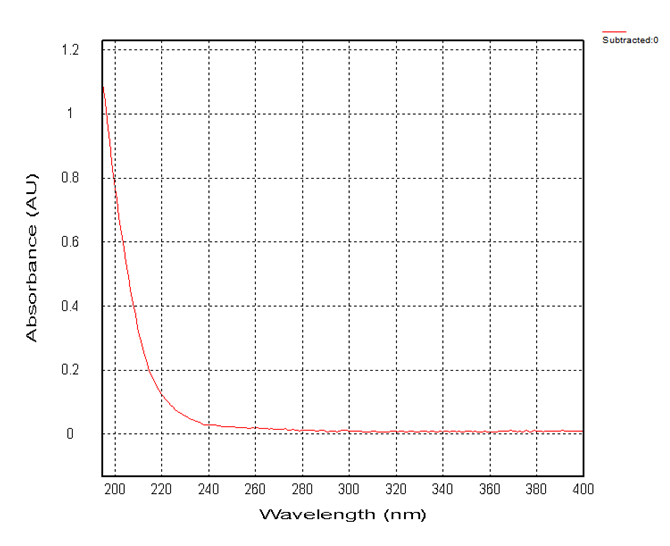


Figure S40. CD (top) and UV (bottom) spectra of artemyrianosin D (**4**)

5. NMR, MS, IR, [*a*]_D_ and CD spectra of compound 5

Figure S41. ^1^H NMR spectrum (600 MHz) of artemyrianosin E (**5**) in CD_3_OD

Figure S42. ^13^C NMR spectrum (150 MHz) of artemyrianosin E (**5**) in CD_3_OD

Figure S43. HSQC spectrum (600 MHz) of artemyrianosin E (**5**) in CD_3_OD

Figure S44. HMBC spectrum (600 MHz) of artemyrianosin E (**5**) in CD_3_OD

Figure S45. ^1^H–^1^H COSY spectrum (600 MHz) of artemyrianosin E (**5**) in CD_3_OD

Figure S46. ROESY spectrum (600 MHz) of artemyrianosin E (**5**) in CD_3_OD


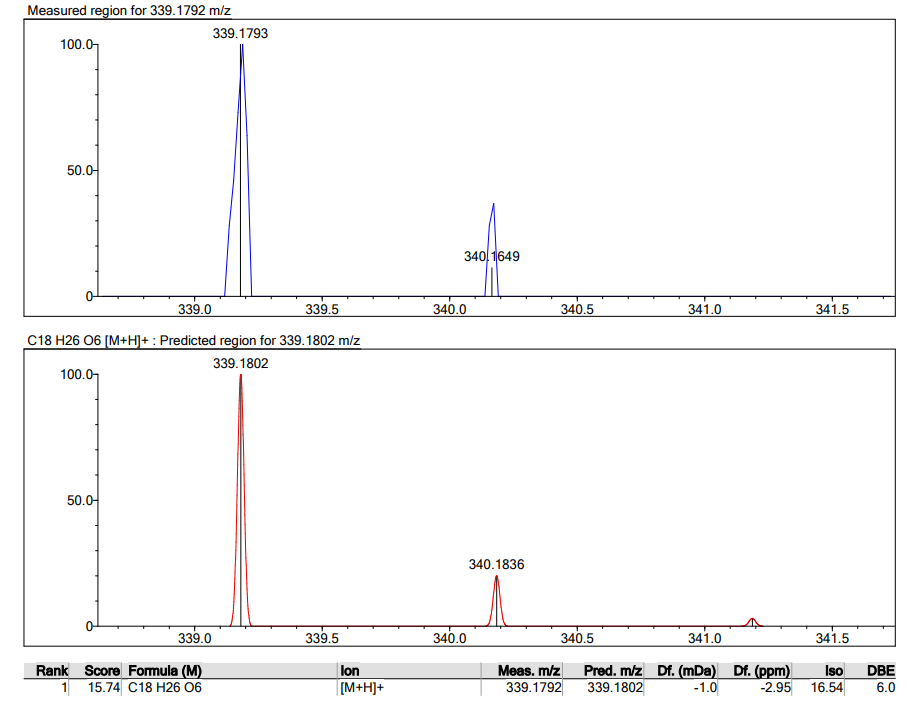


Figure S47. HRESIMS spectrum of artemyrianosin E (**5**)


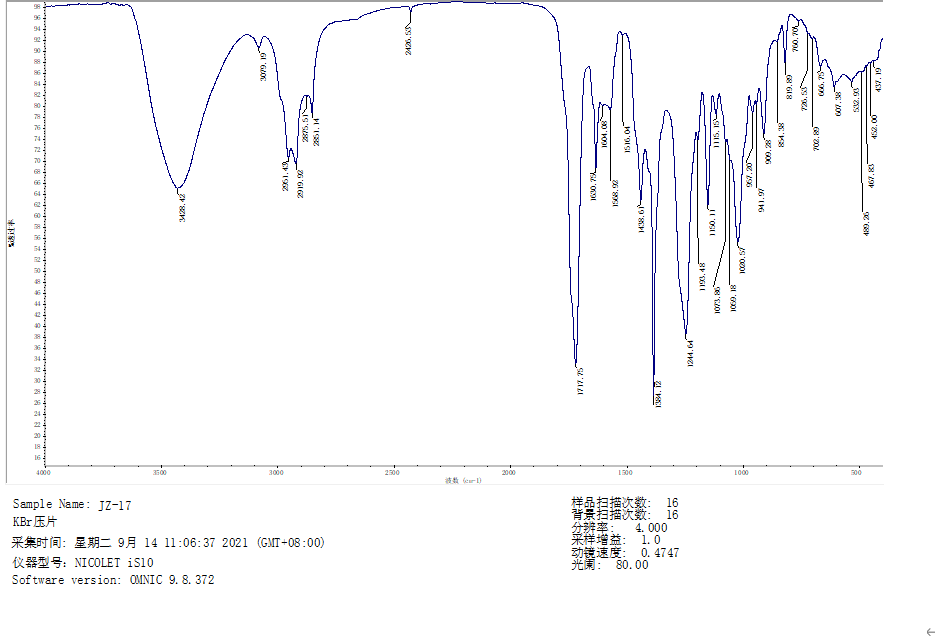


Figure S48. IR spectrum of artemyrianosin E (**5**)


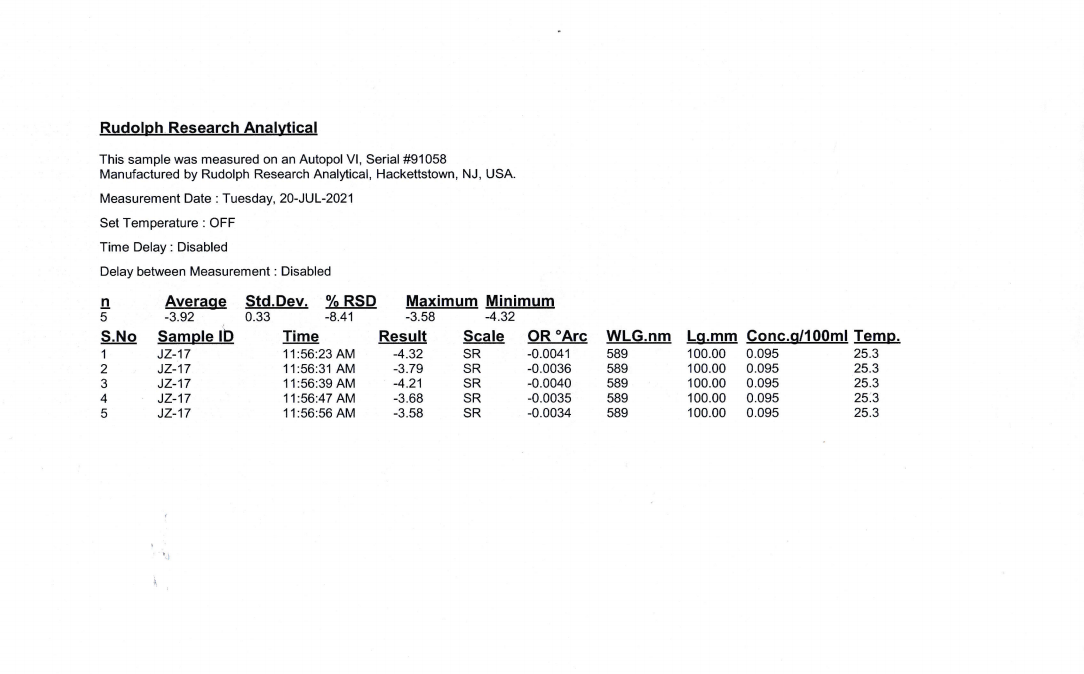


Figure S49. Optical rotation spectrum of artemyrianosin E (**5**)


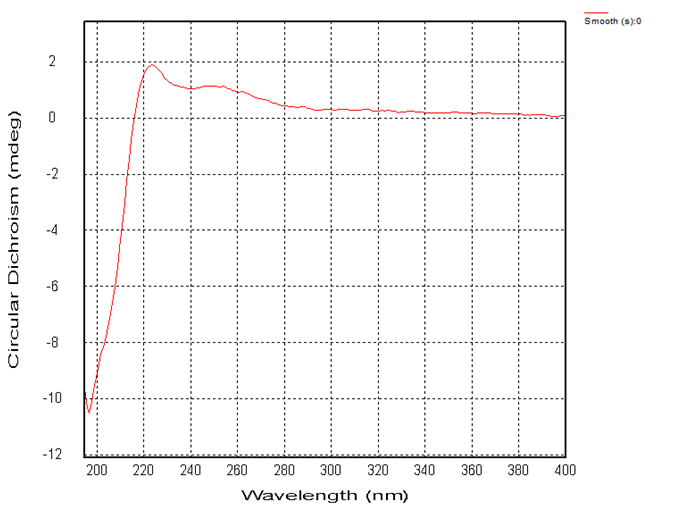

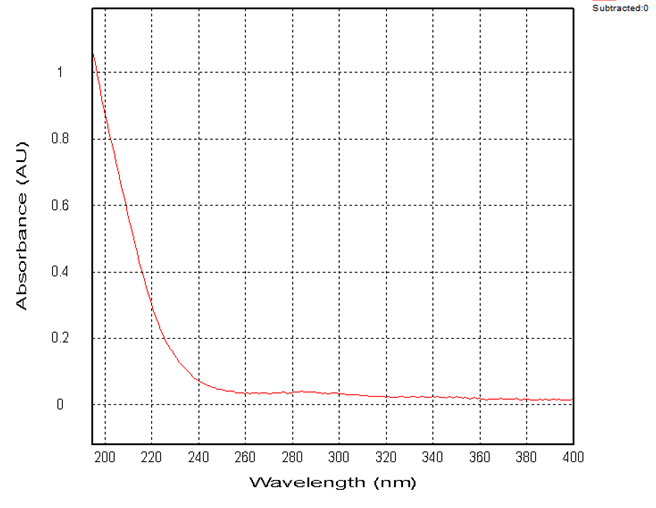


Figure S50. CD (top) and UV (bottom) spectra of artemyrianosin E (**5**)

6. NMR, MS, IR, [*a*]_D_ and CD spectra of compound 6

Figure S51. ^1^H NMR spectrum (600 MHz) of artemyrianosin F (**6**) in CD_3_OD

Figure S52. ^13^C NMR spectrum (150 MHz) of artemyrianosin F (**6**) in CD_3_OD

Figure S53. HSQC spectrum (600 MHz) of artemyrianosin F (**6**) in CD_3_OD

Figure S54. HMBC spectrum (600 MHz) of artemyrianosin F (**6**) in CD_3_OD

Figure S55. ^1^H–^1^H COSY spectrum (600 MHz) of artemyrianosin F (**6**) in CD_3_OD

Figure S56. ROESY spectrum (600 MHz) of artemyrianosin F (**6**) in CD_3_OD


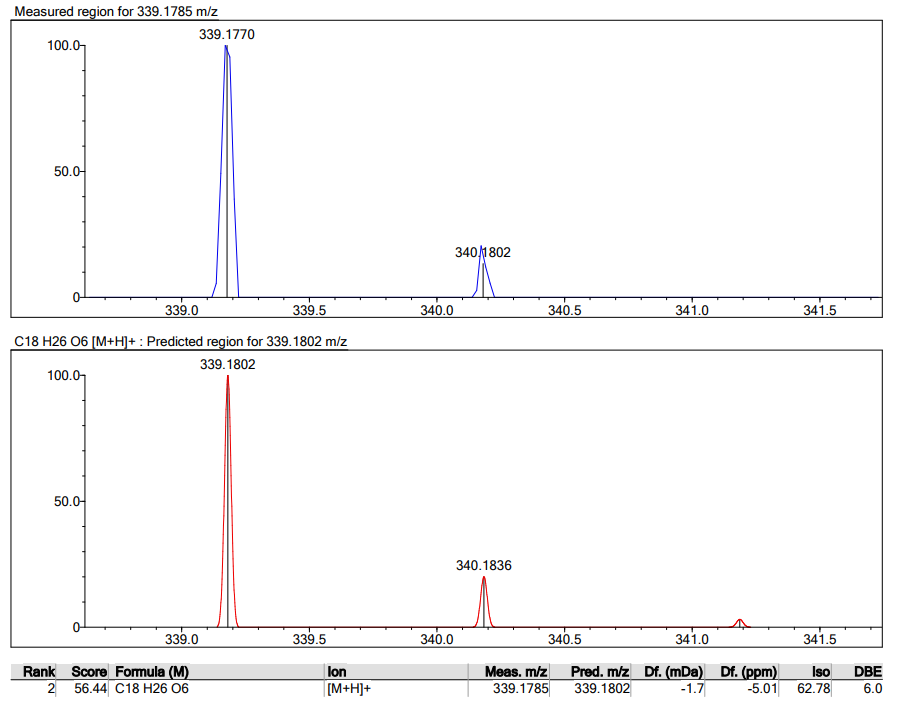


Figure S57. HRESIMS spectrum of artemyrianosin F (**6**)


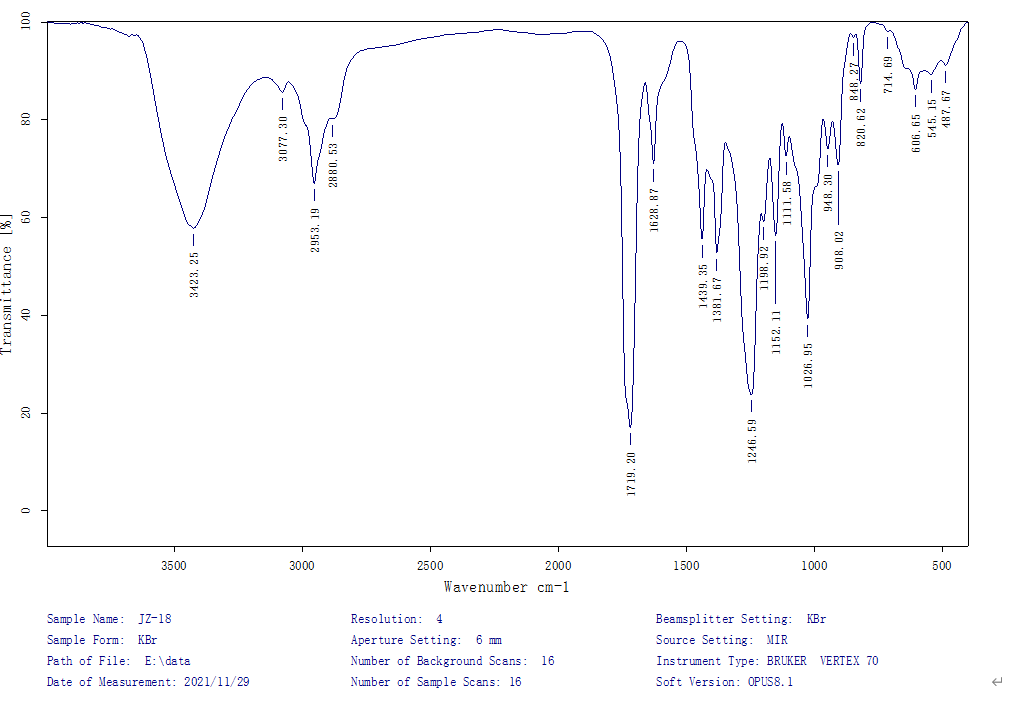


Figure S58. IR spectrum of artemyrianosin F (**6**)


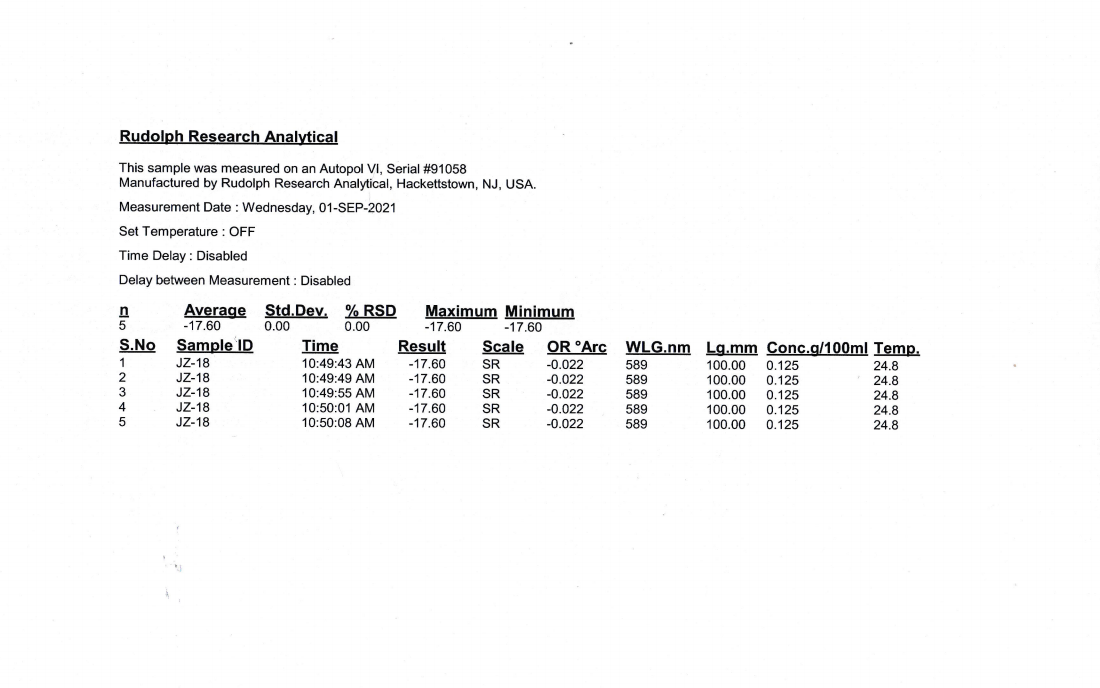


Figure S59. Optical rotation spectrum of artemyrianosin F (**6**)


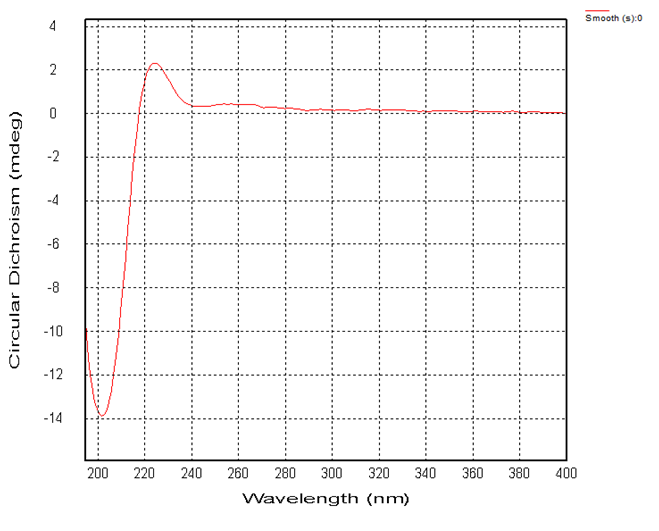

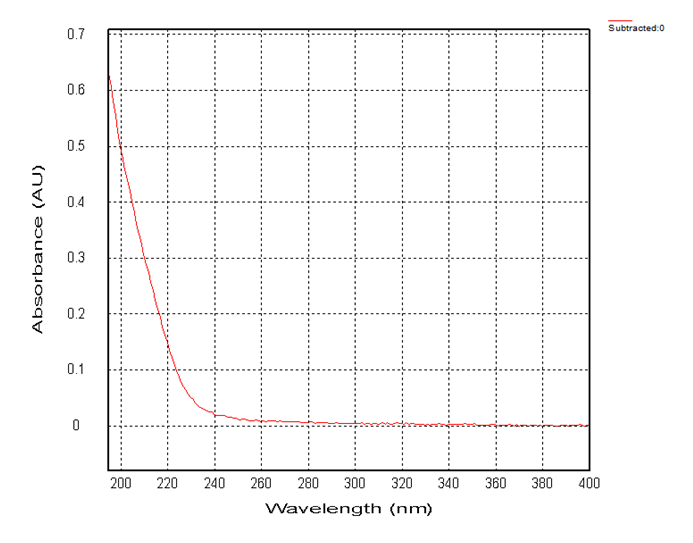


Figure S60. CD (top) and UV (bottom) spectra of artemyrianosin F (**6**)

7. NMR, MS, IR, [*a*]_D_ and CD spectra of compound 7

Figure S61. ^1^H NMR spectrum (600 MHz) of artemyrianosin G (**7**) in CD_3_OD

Figure S62. ^13^C NMR spectrum (150 MHz) of artemyrianosin G (**7**) in CD_3_OD

Figure S63. HSQC spectrum (600 MHz) of artemyrianosin G (**7**) in CD_3_OD

Figure S64. HMBC spectrum (600 MHz) of artemyrianosin G (**7**) in CD_3_OD

Figure S65. ^1^H–^1^H COSY spectrum (600 MHz) of artemyrianosin G (**7**) in CD_3_OD

Figure S66. ROESY spectrum (600 MHz) of artemyrianosin G (**7**) in CD_3_OD


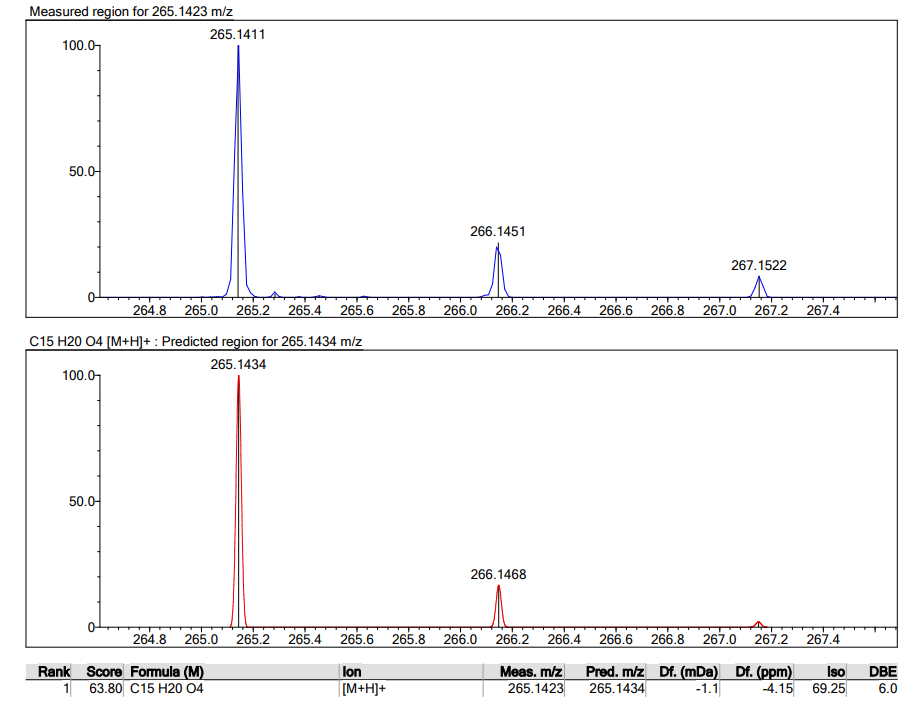


Figure S67. HRESIMS spectrum of artemyrianosin G (**7**)


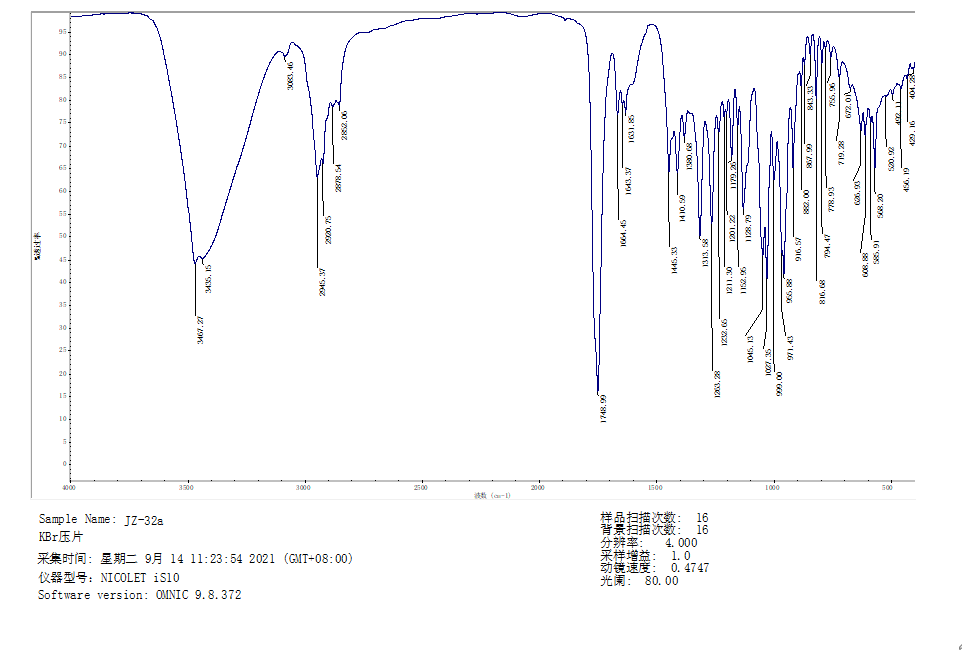


Figure S68. IR spectrum of artemyrianosin G (**7**)


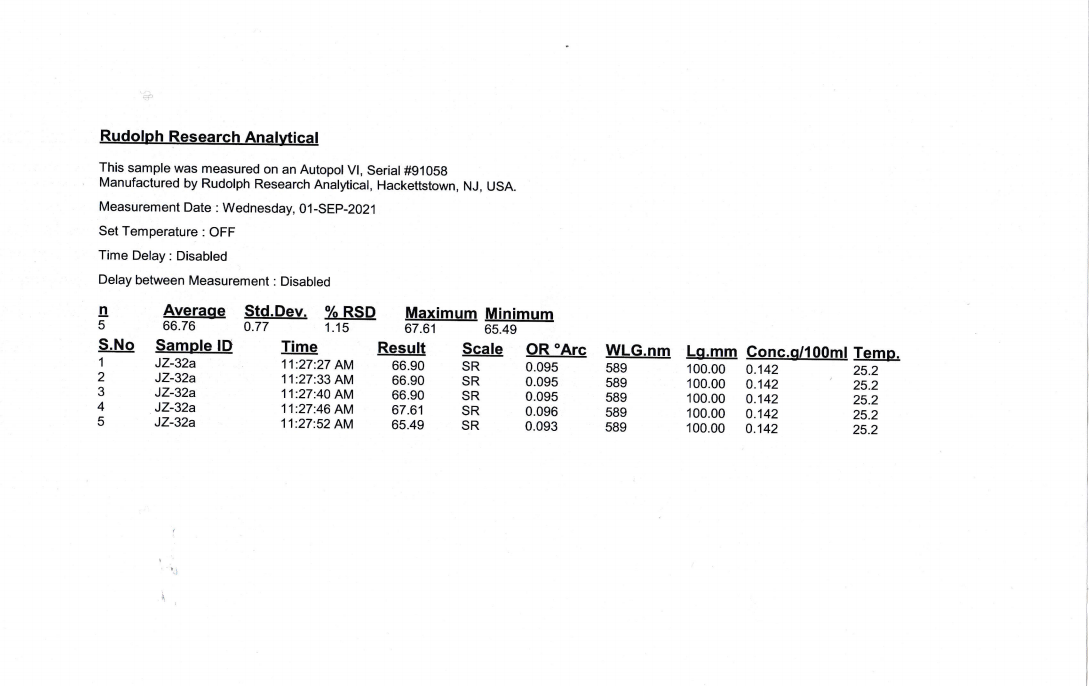


Figure S69. Optical rotation spectrum of artemyrianosin G (**7**)


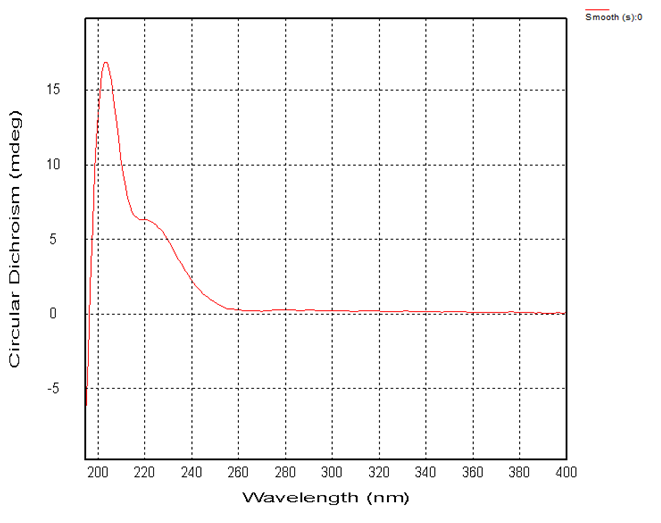

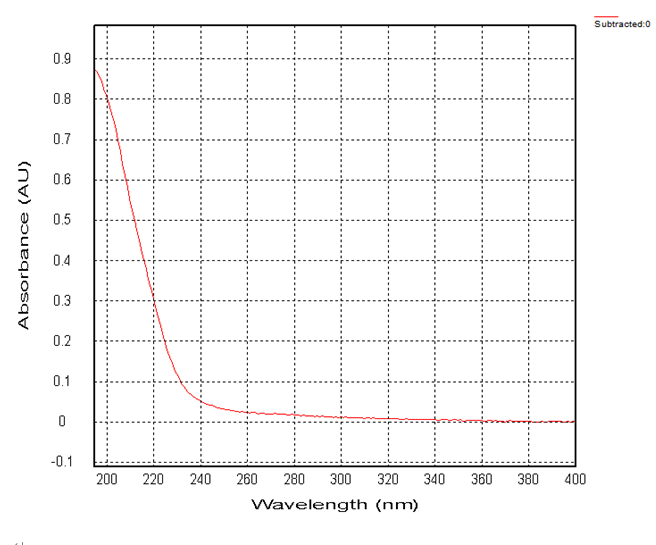


Figure S70. CD (top) and UV (bottom) spectra of artemyrianosin G (**7**)

8. NMR, MS, IR, [*a*]_D_ and CD spectra of compound 8

Figure S71. ^1^H NMR spectrum (600 MHz) of artemyrianosin H (**8**) in CD_3_OD

Figure S72. ^13^C NMR spectrum (150 MHz) of artemyrianosin H (**8**) in CD_3_OD

Figure S73. HSQC spectrum (600 MHz) of artemyrianosin H (**8**) in CD_3_OD

Figure S74. HMBC spectrum (600 MHz) of artemyrianosin H (**8**) in CD_3_OD

Figure S75. ^1^H–^1^H COSY spectrum (600 MHz) of artemyrianosin H (**8**) in CD_3_OD

Figure S76. ROESY spectrum (600 MHz) of artemyrianosin H (**8**) in CD_3_OD


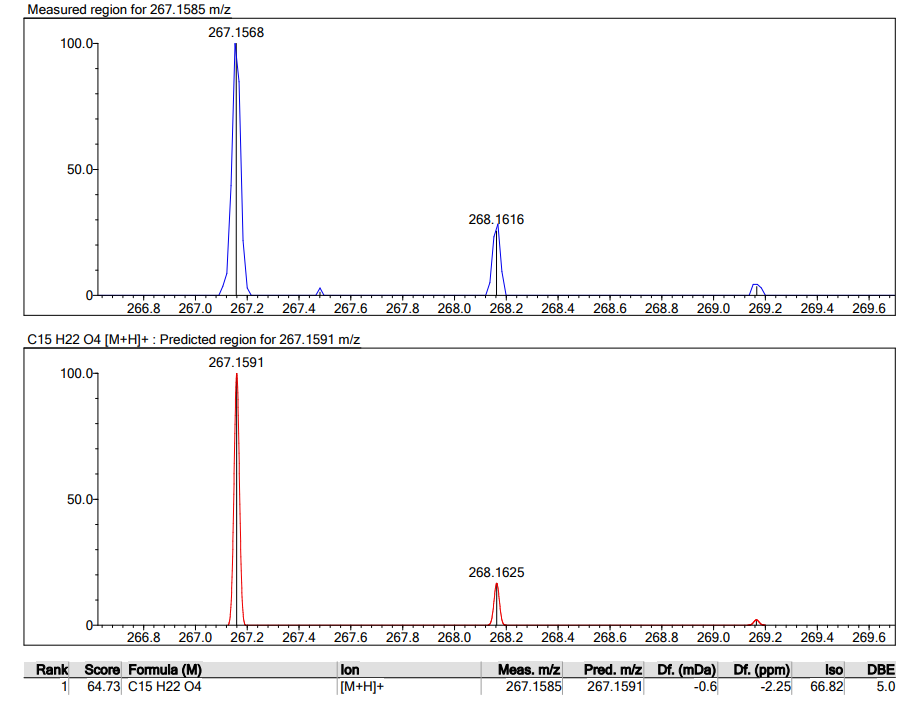


Figure S77. HRESIMS spectrum of artemyrianosin H (**8**)


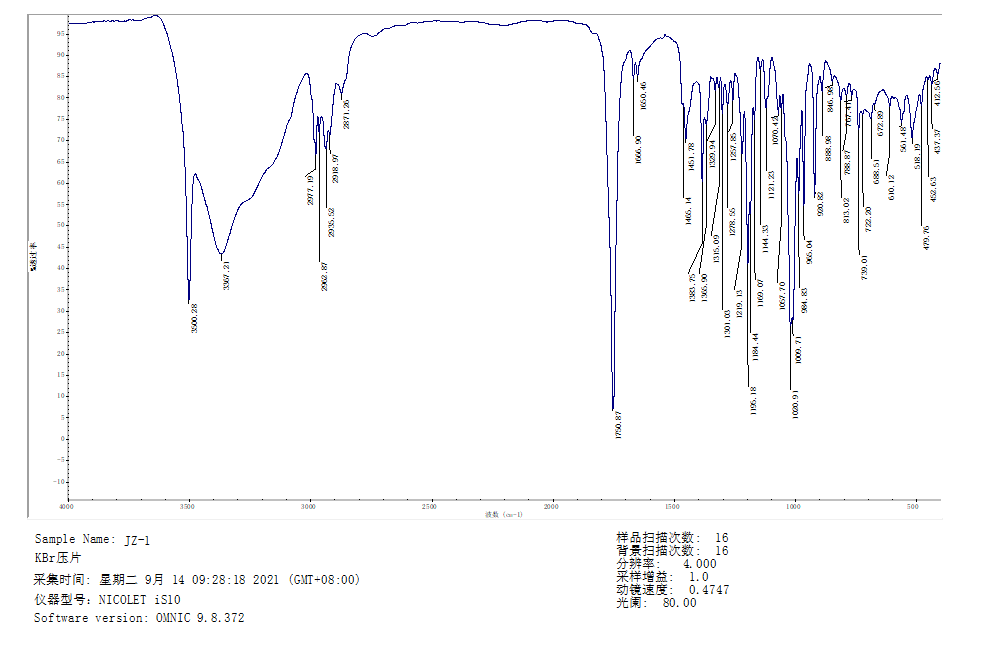


Figure S78. IR spectrum of artemyrianosin H (**8**)


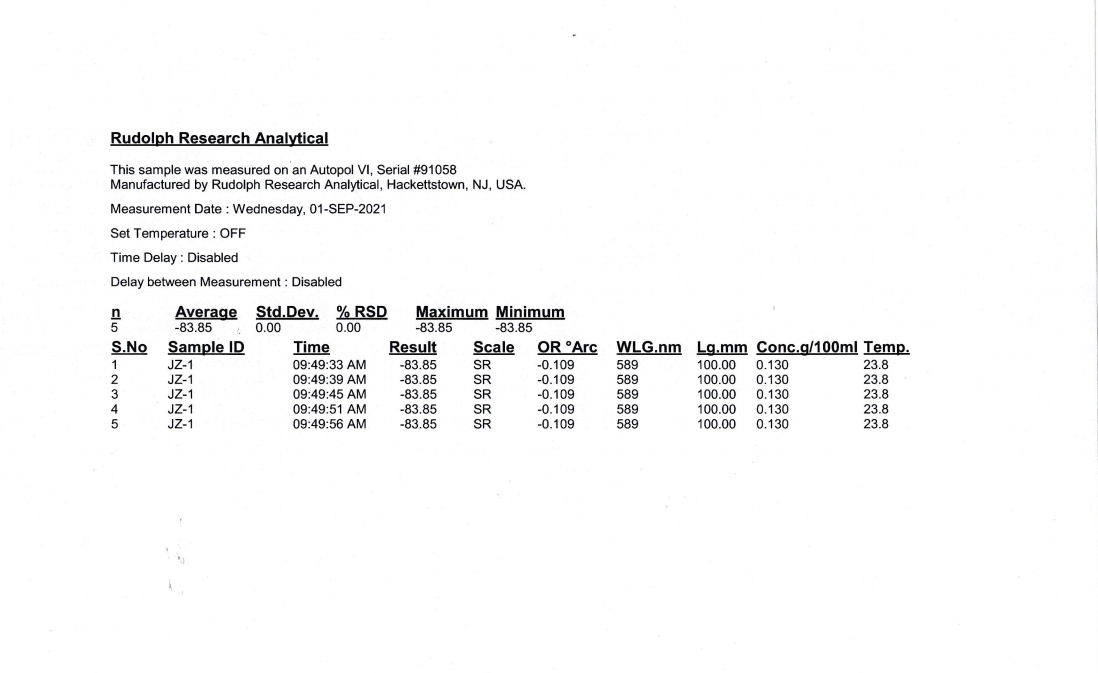


Figure S79. Optical rotation spectrum of artemyrianosin H (**8**)


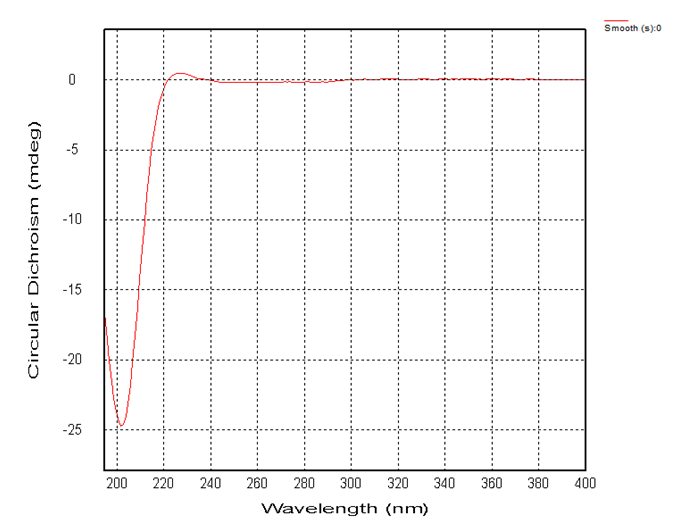

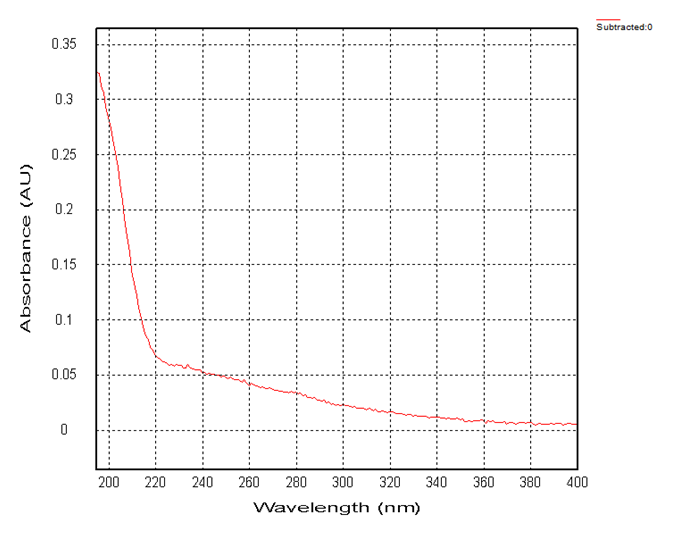


Figure S80. CD (top) and UV (bottom) spectra of artemyrianosin H (**8**)

9. NMR, MS, IR, [*a*]_D_ and CD spectra of compound 9

Figure S81. ^1^H NMR spectrum (600 MHz) of artemyrianosin I (**9**) in CD_3_OD

Figure S82. ^13^C NMR spectrum (150 MHz) of artemyrianosin I (**9**) in CD_3_OD

Figure S83. HSQC spectrum (600 MHz) of artemyrianosin I (**9**) in CD_3_OD

Figure S84. HMBC spectrum (600 MHz) of artemyrianosin I (**9**) in CD_3_OD

Figure S85. ^1^H–^1^H COSY spectrum (600 MHz) of artemyrianosin I (**9**) in CD_3_OD

Figure S86. ROESY spectrum (600 MHz) of artemyrianosin I (**9**) in CD_3_OD


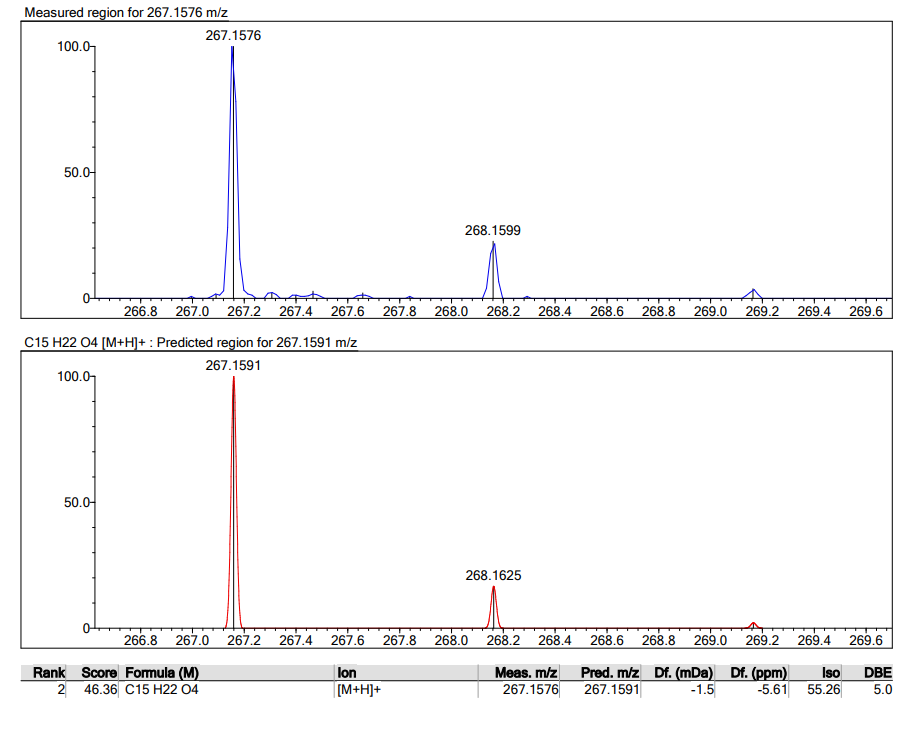


Figure S87. HRESIMS spectrum of artemyrianosin I (**9**)


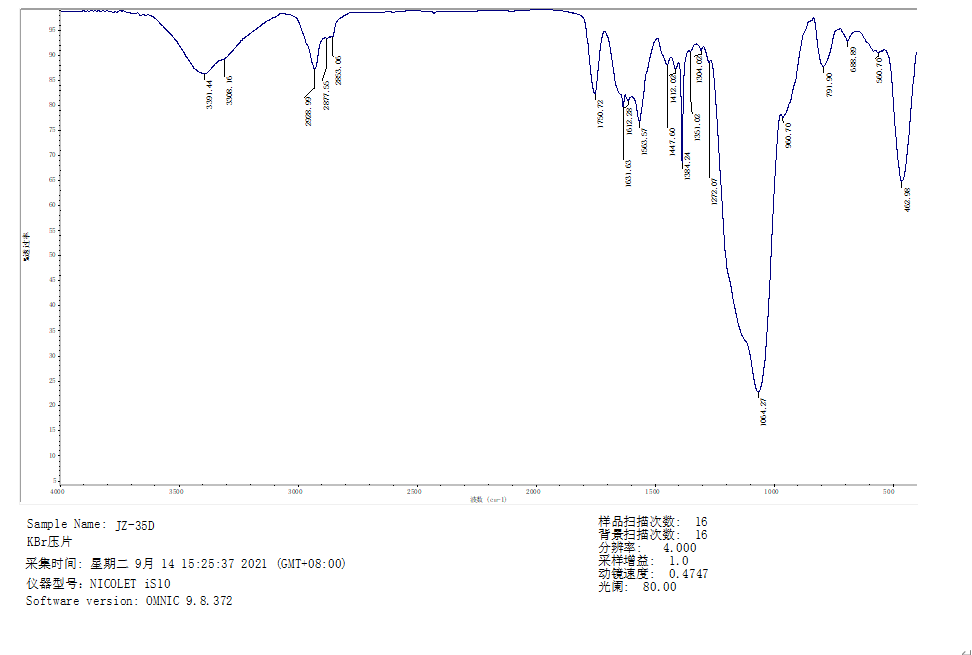


Figure S88. IR spectrum of artemyrianosin I (**9**)


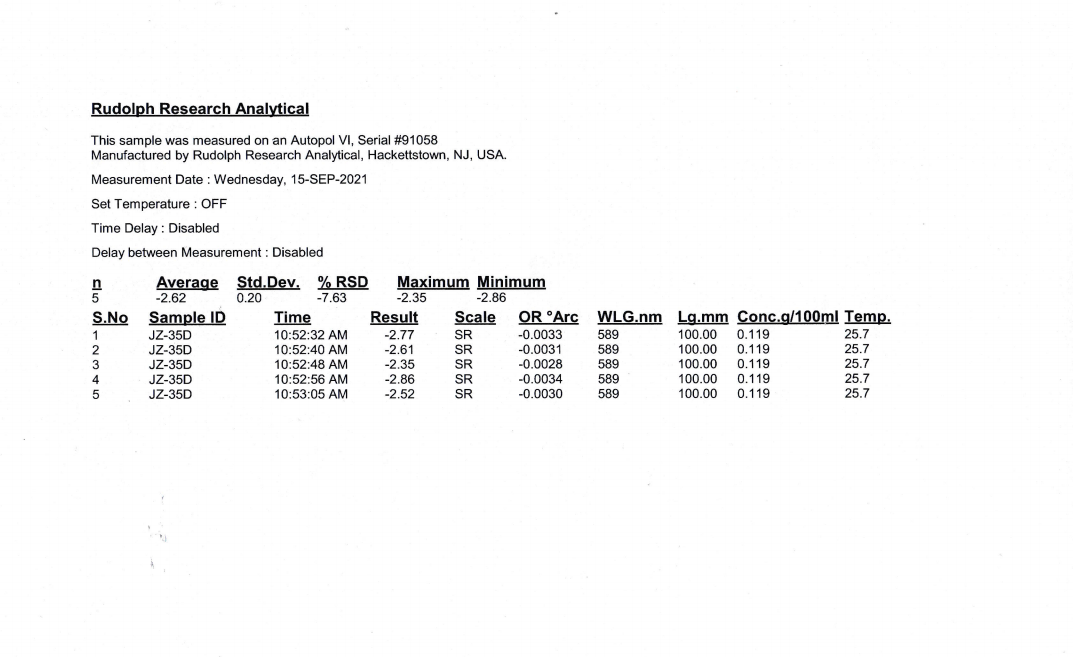


Figure S89. Optical rotation spectrum of artemyrianosin I (**9**)


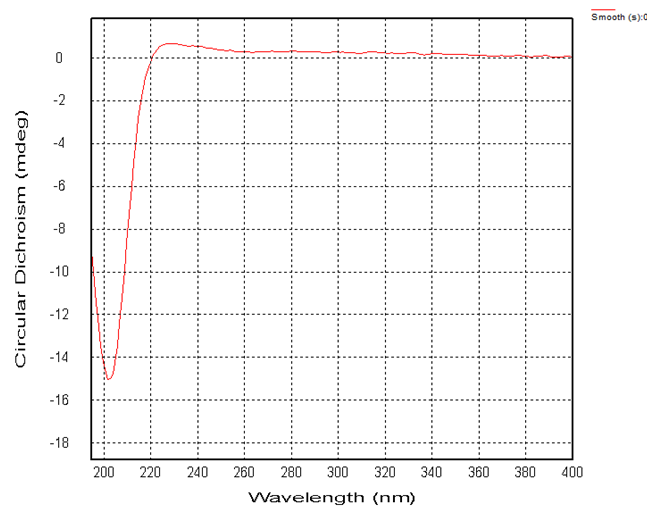

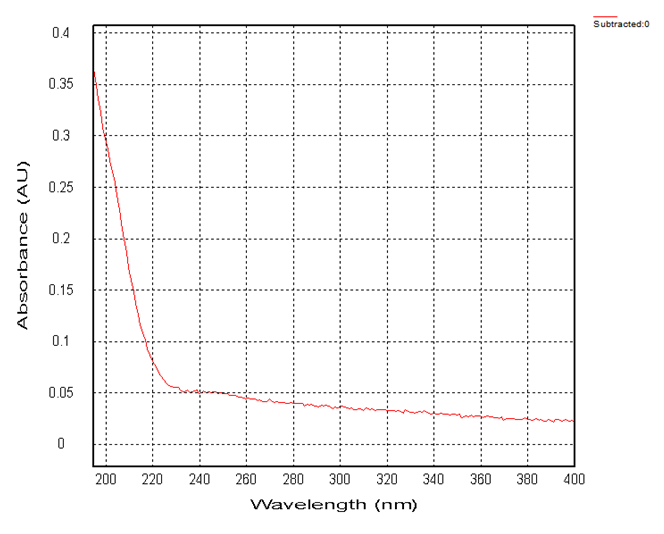


Figure S90. CD (top) and UV (bottom) spectra of artemyrianosin I (**9**)

13. NMR, MS, IR, [*a*]_D_ and CD spectra of compound 10

Figure S91. ^1^H NMR spectrum (600 MHz) of artemyrianosin J (**10**) in CD_3_OD

Figure S92. ^13^C NMR NMR spectrum (150 MHz) of artemyrianosin J (**10**) in CD_3_OD

Figure S93. HSQC spectrum (600 MHz) of artemyrianosin J (**10**) in CD_3_OD

Figure S94. HMBC spectrum (600 MHz) of artemyrianosin J (**10**) in CD_3_OD

Figure S95. ^1^H–^1^H COSY spectrum (600 MHz) of artemyrianosin J (**10**) in CD_3_OD

Figure S96. ROESY spectrum (600 MHz) of artemyrianosin J (**10**) in CD_3_OD


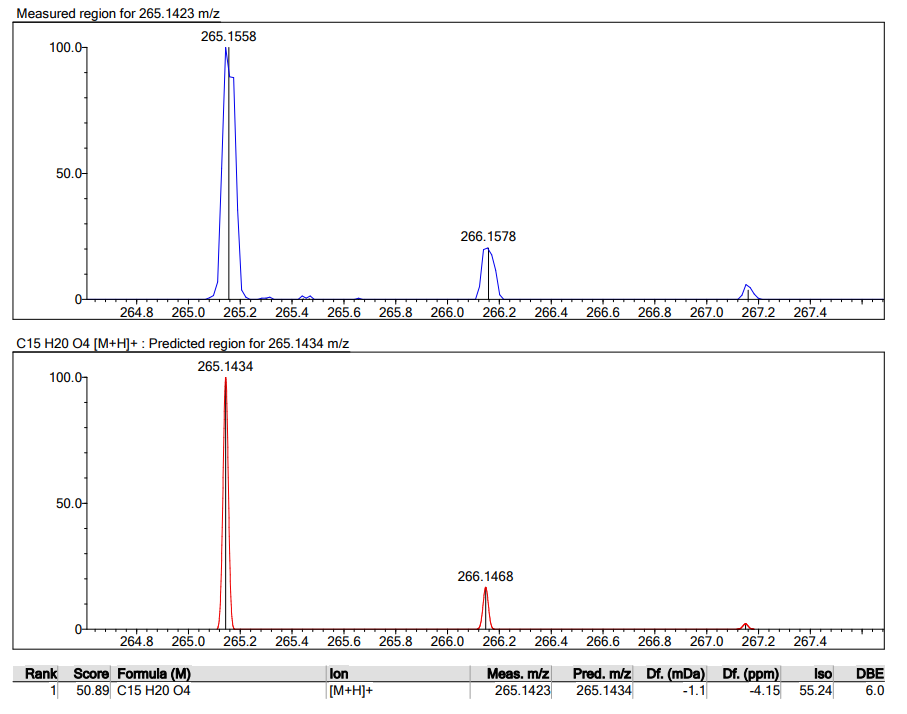


Figure S97. HRESIMS spectrum of artemyrianosin J (**10**)


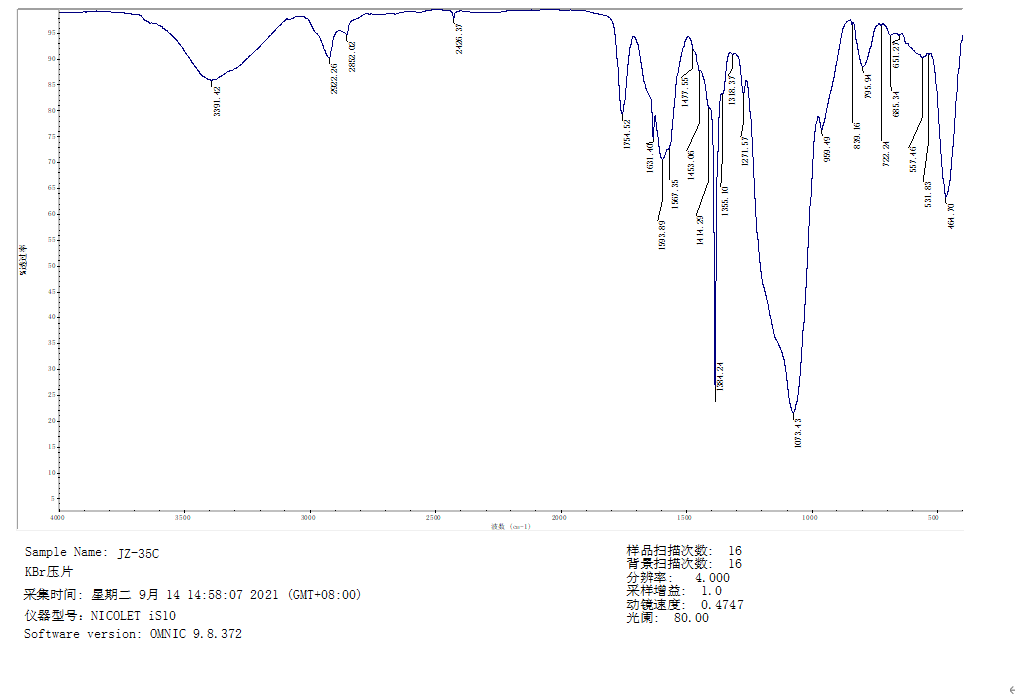


Figure S98. IR spectrum of artemyrianosin J (**10**)


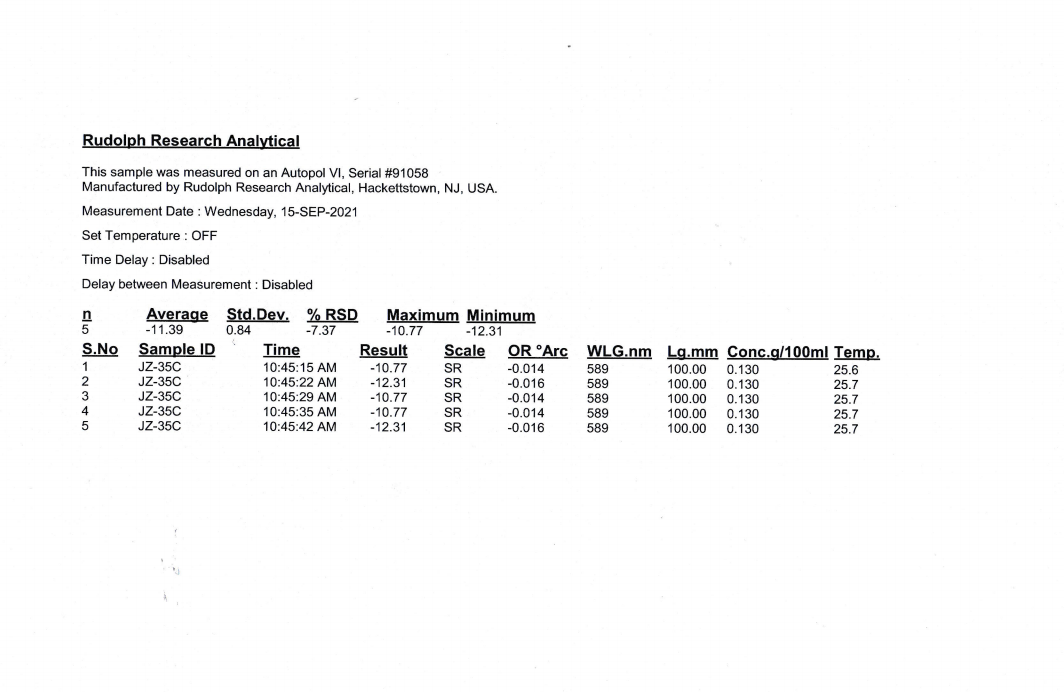


Figure S99. Optical rotation spectrum of artemyrianosin J (**10**)


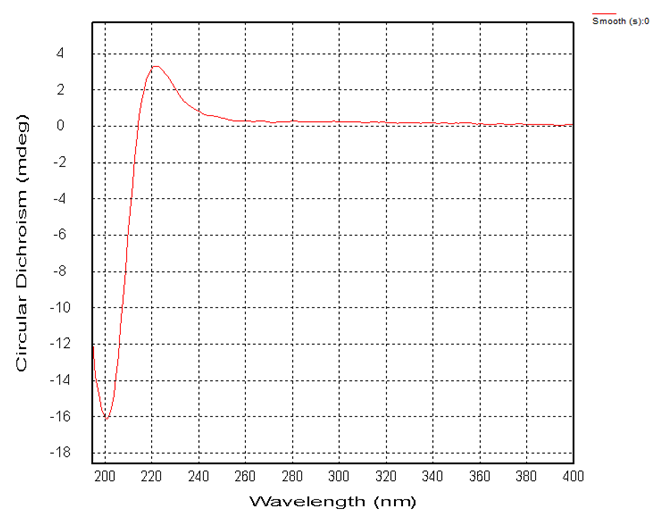

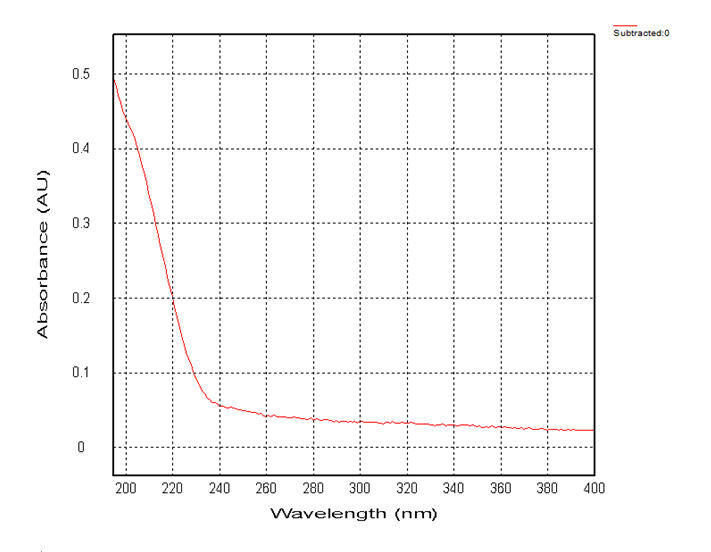


Figure S100. CD (top) and UV (bottom) spectra of artemyrianosin J (**10**)
